# Synthesis, Immunosuppressive Properties, and Mechanism of Action of a New Isoxazole Derivative

**DOI:** 10.3390/molecules23071545

**Published:** 2018-06-26

**Authors:** Marcin Mączyński, Sylwia Borska, Katarzyna Mieszała, Maja Kocięba, Ewa Zaczyńska, Iwona Kochanowska, Michał Zimecki

**Affiliations:** 1 Faculty of Pharmacy, Department of Organic Chemistry, Wroclaw Medical University, Borowska 211A, 50-556 Wroclaw, Poland; 2 Faculty of Medicine, Histology and Embryology Division, Wroclaw Medical University, Chalubinskiego 6a, 50-368 Wroclaw, Poland; sylwia.borska@umed.wroc.pl (S.B.); katarzynamieszala@wp.pl (K.M.); 3 Institute of Immunology and Experimental Therapy, Laboratory of Immunobiology, Weigla 12, 53-114 Wroclaw, Poland; kocieba@iitd.pan.wroc.pl (M.K.); ezacz@iitd.pan.wroc.pl (E.Z.); kochanie@iitd.pan.wroc.pl (I.K.); zimecki@iitd.pan.wroc.pl (M.Z.)

**Keywords:** isoxazole, PBMC, proliferation, TNF α, apoptosis, jurkat cells

## Abstract

This work describes the synthesis of a new series of isoxazole derivatives, their immunosuppressive properties, and the mechanism of action of a representative compound. A new series of *N*′-substituted derivatives of 5-amino-*N*,3-dimethyl-1,2-oxazole-4-carbohydrazide (**MM1**–**MM10**) was synthesized in reaction of 5-amino-*N*,3-dimethyl-1,2-oxazole-4-carbohydrazide with relevant carbonyl compounds. The isoxazole derivatives were tested in several in vitro models using human cells. The compounds inhibited phytohemagglutinin A (PHA)-induced proliferation of peripheral blood mononuclear cells (PBMCs) to various degrees. The toxicity of the compounds with regard to a reference A549 cell line was also differential. 5-amino-*N*′-(2,4-dihydroxyphenyl)methylidene-*N*,3-dimethyl-1,2-oxazole-4-carbohydrazide (**MM3**) compound was selected for further investigation because of its lack of toxicity and because it had the strongest antiproliferative activity. The compound was shown to inhibit lipopolysaccharide (LPS)-induced tumor necrosis factor (TNF α) production in human whole blood cell cultures. In the model of Jurkat cells, **MM3** elicited strong increases in the expression of caspases, Fas, and NF-κB1, indicating that a proapoptotic action may account for its immunosuppressive action in the studied models.

## 1. Introduction

Isoxazoles are an important class of heterocyclic compound, displaying a broad spectrum of biological activities. Modification in their structures has offered a high degree of diversity that has proved useful for the development of new therapeutic agents with improved potency and lower toxicity. Isoxazole derivatives display a wide array of pharmacological activities that have been successfully screened for anticancer [1], anti-inflammatory [2], antimicrobial [3], antihistaminic, antitubercular, antiulcer [4], antiepileptic [5], dual α2-adrenoreceptor and 5-HTreuptekeinhibitors [6], antiviral [7], and anxiolytic [8] activities. The pharmacological profits of employing the isoxazole ring are due to the fact that this structure acts as a key pharmacophore for the biological activity of such drugs as Valdecoxib (COX-2 inhibitor) [9] and Leflunomide (antirheumatic drug) [10]. The compounds containing the isoxazole ring are also promising therapeutic agents in neurodegenerative diseases because of their similarity to the α-amino-3-hydroxy-5-methyl-4-isoxazolepropionic acid, which is a specific agonist for the α-amino-3-hydroxy-5-methyl-4-isoxazolepropionic acid receptor (AMPA) receptor [11].

Over the last few decades, non-toxic, low molecular weight, and selectively acting compounds containing the isoxazole moiety were synthesized by Ryng and Mączyński, and their immunological activities were reported [12,13,14,15]. Our previous studies described the immuno-stimulatory [16,17], immuno-restituting [18,19,20], immunosuppressive [13,17,21], and anti-inflammatory [22] activities of the isoxazole derivatives. Interestingly, isoxazole derivatives differing only in one substituent could exhibit opposite immunological functions [23]. The best results in designing new immunomodulators within the isoxazole family are achieved by structural modifications of the previously synthesized compounds of well-established activity. Interestingly, among all the compounds synthesized by our team, the modification in position 4 of the 5-amino-3-methyl-4-isoxazolecarboxylic acid led to unexpected biological activity. The mono-substituted 5-amino-3-methyl-4-isoxazolecarboxylic acid phenylamides with immune-stimulatory activity equal to or higher than levamisole (a reference drug) were described [17]. Further characterization of these compounds revealed their very low toxicity. Compounds substituted in position 4 with a strong electrophilic group were most active. 4-chlorophenylamide of 5-amino-3-methyl-4-isoxazolecarboxylic acid exerted a strong stimulating effect on the humoral and cellular immune responses [24,25,26]. On the other hand, di- and tri-substituted 5-amino-3-methyl-4-isoxazolecarboxylic acid phenylamides showed immunosuppressive activity more effectively than cyclosporine A, which was used as a reference drug. Dichloro- and trimethoxy-derivatives of 5-amino-3-methyl-4-isoxazolecarboxylic acid phenylamides were inhibitors of the humoral immune response [12,25]. 5-amino-3-methyl-4-isoxazolecarboxylic acid benzylamides, substituted in the phenyl ring, presented anti-proliferative and anti-inflammatory properties in vitro and in vivo, in mouse and human models with no apparent toxicity against these cells. 5-amino-3-methyl-*N*-(4-methylbenzyl)-4-isoxazolecarboxamide, selected as the most interesting compound, exhibited strong anti-proliferative and anti-inflammatory properties. On the other hand, this compound, had the ability to enhance manifestation of the cellular immune response [27]. 5-amino-3-methyl-4-isoxazolecarboxylic acid semicarbazides and thiosemicarbazides were also synthesized and tested for their immunological activity. The compounds demonstrated dose-dependent actions ranging from immunosuppressive to immunostimulatory [28,29,30]. *N*′-substituted derivatives of 5-amino-3-methyl-1,2-oxazole-4-carbohydrazide exhibited, in turn, other immunosuppressive activity in mouse and human experimental models [14]. The described results showed that many of the compounds containing isoxazole moiety exhibited immunosuppressive effects on phytohemagglutinin-induced peripheral blood mononuclear cell (PBMC) proliferation and several biological activities and that the isoxazole structure was responsible for the immunological activity [31]. 

Differential and interesting activities of described compounds prompted us to continue modifications of position 4 of the isoxazole moiety. The aim of our study was to synthesize a new series of *N*′-substituted derivatives of 5-amino-*N*,3-dimethyl-1,2-oxazole-4-carbohydrazide in the reaction of 5-amino-*N*,3-dimethyl-1,2-oxazole-4-carbohydrazide with relevant carbonyl compounds and to evaluate their immunosuppressive properties using human blood cells in selected models. Further, we investigated the molecular basis of the immunosuppressive activity of the most active compound using Jurkat cells. 

## 2. Results

### 2.1. Chemistry

**Scheme 1:**
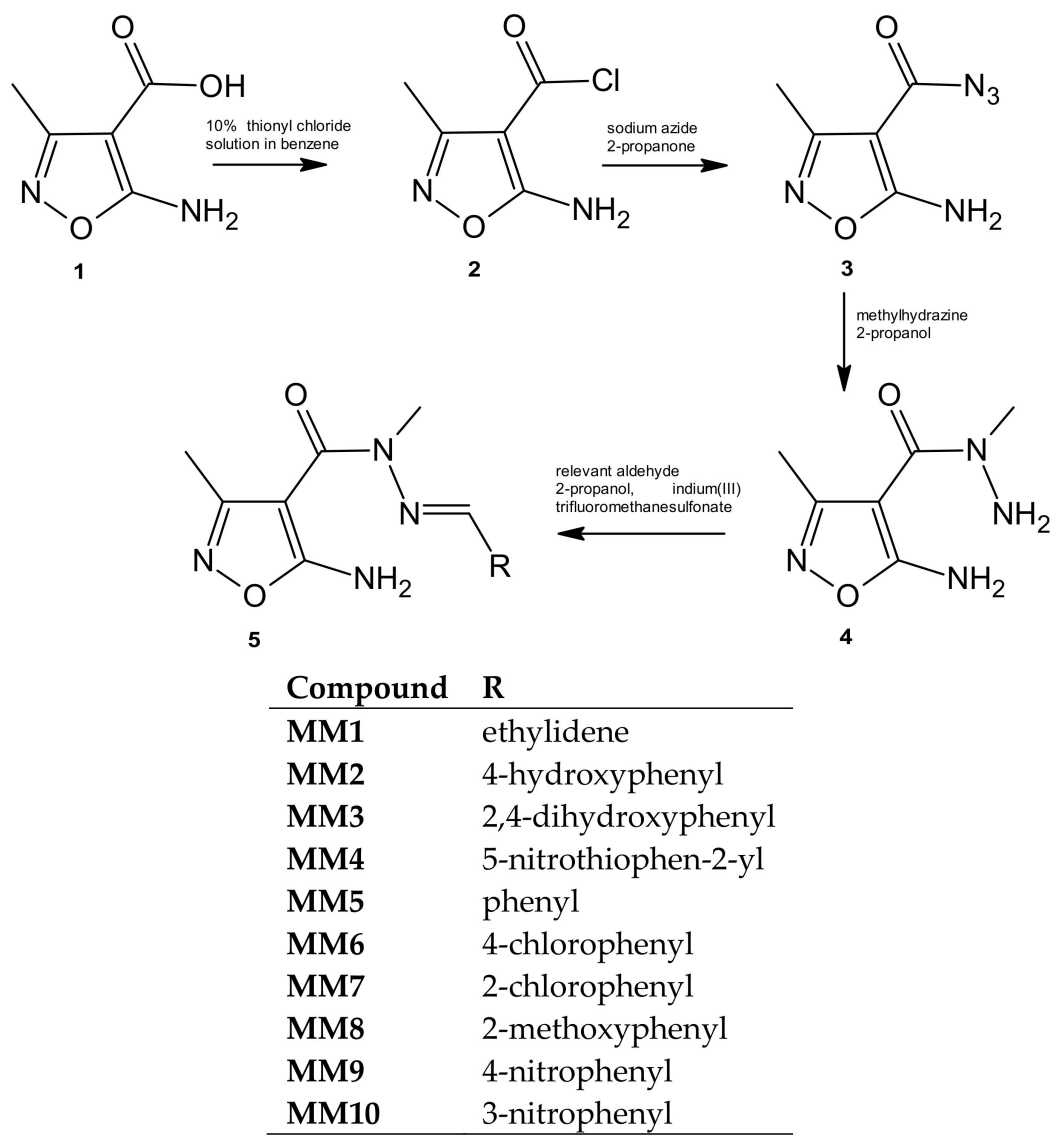
Synthesis of *N*′-substituted derivatives of 5-amino-*N*,3-dimethyl-1,2-oxazole-4-carbohydrazide-**MM1**–**10** compounds.

A series of *N*′-substituted derivatives of 5-amino-*N*,3-dimethyl-1,2-oxazole-4-carbohydrazide-**MM1**–**10** (compounds **5** in Scheme 1) was synthesized according to the synthetic pathway presented in Scheme 1.

The following reagents and substrates were used in the synthesis of the considered compounds. Starting materials, such as esters of 2-cyano-3-alkoxy-2-butenoate acids and semi-products (i.e., known ethyl [32] or methyl [33] esters of 5-amino-3-methyl-4-isoxazolecarboxylic acid), were prepared according to the highly efficient, environmentally friendly methods precisely described in [34,35] and [patent priority number PL20110397559, PL 216764 B1 20140530, PL 216770 B1 20140530]. Known 5-amino-3-methyl-4-isoxazolecarboxylic acid (compound **1**, Scheme 1) was obtained by hydrolysis of its appropriate ethyl or methyl esters with a boiling water solution of sodium hydroxide.

Initially, compounds **2**–**3** were obtained using the method described earlier [patent priority number PL 193939] and were used to obtain compounds **4** and **5** (Scheme 1). Final compound **5** (Scheme 1) was synthesized in reaction with the nucleophilic addition of a primary amine group (terminal group of 5-amino-*N*,3-dimethyl-1,2-oxazole-4-carbohydrazide) with appropriate aromatic aldehyde using indium (III) trifluoromethanesulfonate as a catalyst. The amine group in position 5 in the isoxazole ring is not active in the condition of a realized reaction. The product received imine derivatives (**MM1**–**10**). The applied method provided products in good yields of up to 63–81%. The structures of the obtained compounds **MM1**–**10** were characterized by a sharp melting point (m.p.), IR, ^1^H NMR spectra, and MS.

### 2.2. Biology

**Figure 1:**
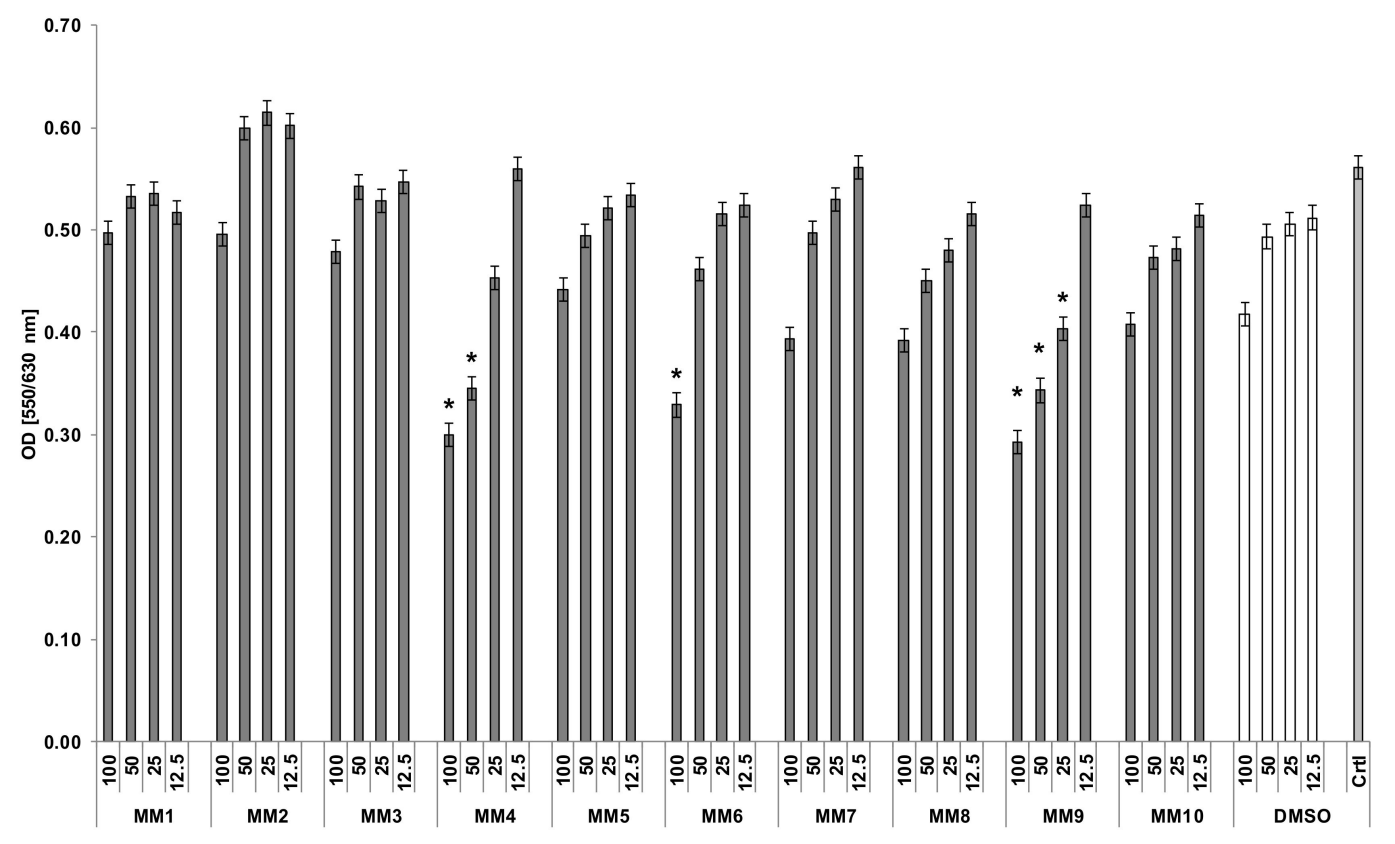
The toxicity of the compounds against the A549 cell line.

In pilot experiments (Supplementary Materials, Figure S3), we found that compounds **MM2**, **MM3**, **MM5**, **MM6**, and **MM7** inhibited to various degrees concanavalin A-induced proliferation of mouse splenocytes. The best inhibitory effects were observed with **MM3**, but **MM2** and **MM7** were also strongly suppressive. The determination of the cytotoxicity in relation to the L929 cell line did not reveal toxic effects of the compounds (Supplementary Materials, Figure S1). However, cytotoxic actions of some compounds (**MM4**, **MM6**, and **MM9**) could be demonstrated at a higher concentration by using the A549 reference cell line (Figure 1). **MM3**, in turn, appeared to be devoid of cell toxicity until a tested concentration of 250 μM (Supplementary Materials, Figure S2). 

The cytotoxicity of the compounds was determined by measuring the growth of the human tumor epithelial lung A549 cell line. The results are presented as optical density (OD) values. Statistics are presented as *p* < 0.05 versus appropriate dilutions of the solvent (DMSO).

**Figure 2:**
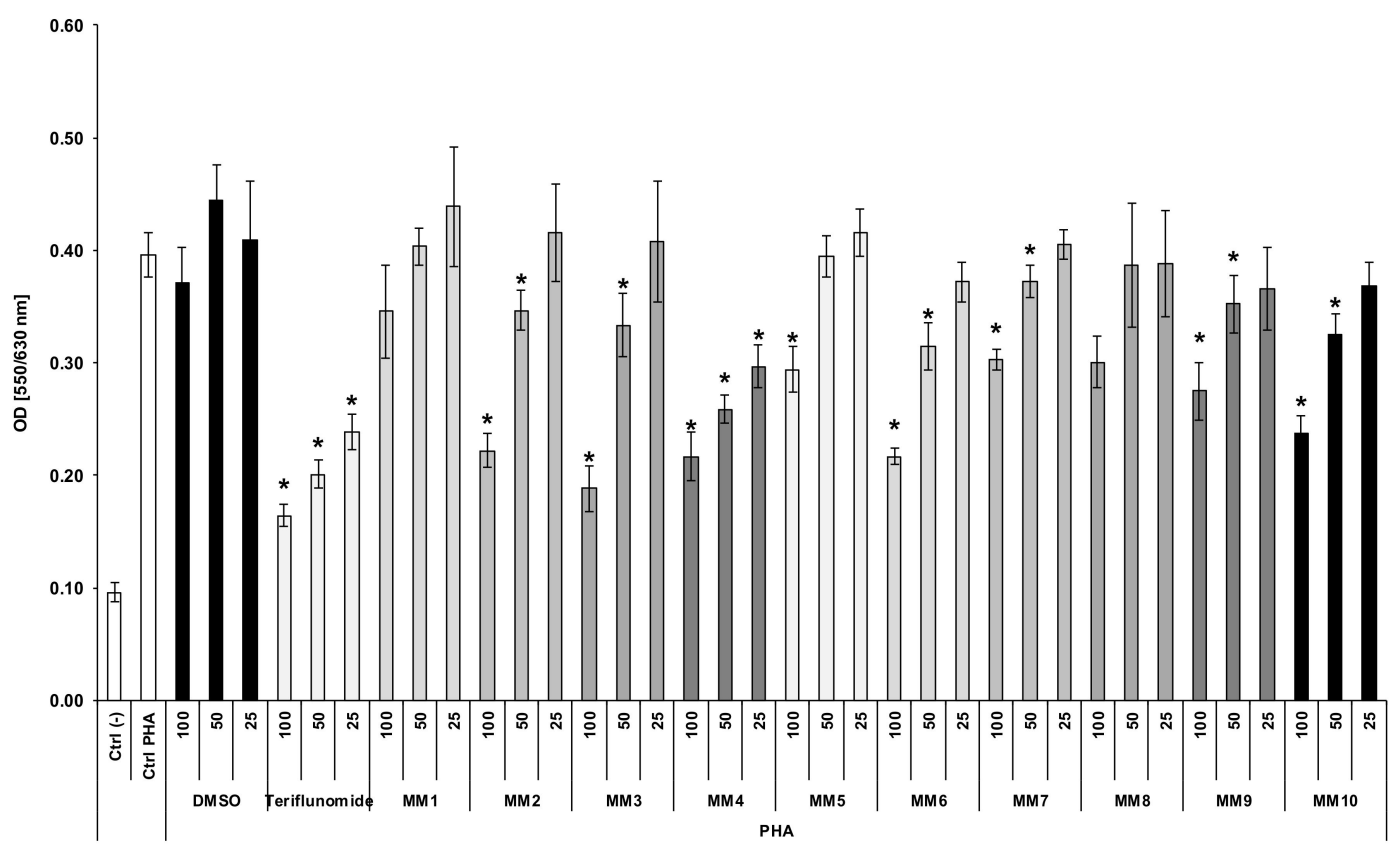
Effects of the MM compounds on phytohemagglutinin A (PHA)-induced peripheral blood mononuclear cell (PBMC) proliferation.

The results showed that the compounds exhibited differential abilities to suppress phytohemagglutinin A (PHA)-induced lymphocyte proliferation (Figure 2). The most suppressive was **MM3**, as in the case of concanavalin-induced splenocyte proliferation (Supplementary Materials, Figure S3). Its inhibitory action, in terms of kinetics, resembled that of teriflunomide but was somewhat weaker.

**Figure 3:**
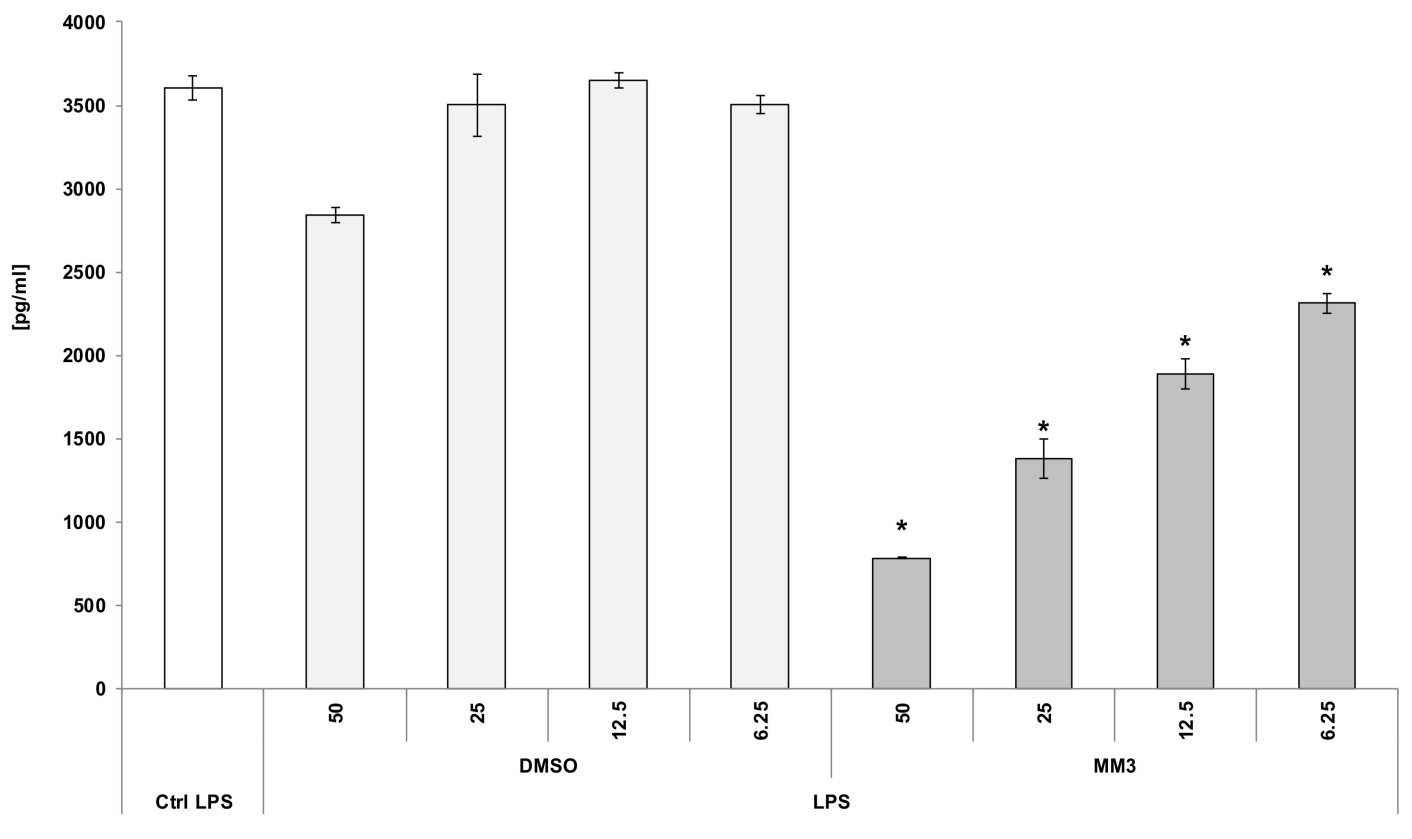
Effect of **MM3** compound on lipopolysaccharide (LPS)-induced tumor necrosis factor (TNF α) production in whole blood culture. * *p* < 0.05 versus appropriate dilutions of the solvent (DMSO).

**MM3** inhibited, in a dose-dependent manner, inducible tumor necrosis factor (TNF α) production (Figure 3). The inhibition was still significant at a concentration of 6.25 μM (about 40% inhibition). The interdependence between activities, toxicity, and selected biological activities are presented in Table 1*.*

**MM3** compound was incubated for 24 h at a concentration of 50 μM with Jurkat cells, and the changes in the expression of signaling molecules were measured as described in Section 4. The results (Table 2) demonstrated strong upregulation of caspase expression, as well as NF-κB1 and Fas signaling proteins. No increase of p53 and a negligible change in Bcl-2 expression was noted.

## 3. Discussion

In this investigation, we evaluated the ability of the **MM1**–**10** compounds and subsequently, that of **MM3**, to suppress proliferation of mitogen-induced human blood lymphocytes and TNF α production by human blood cultures. Teriflunomide, an isoxazole drug [36], served as a reference compound in the proliferation test. **MM3** was not toxic with regard to the reference cell line A549 and, up to the studied concentration of 250 μM, showed a moderate inhibition of the cell proliferation but a strong, dose-dependent suppression of TNF α production. 

The design of a compound’s structure with a more beneficial therapeutic property and less toxicity than a leading structure is a key task in the search for new potential drugs. In the described **MM1**–**10** series, 5-amino-*N*,3-dimethyl-*N*′-phenylmethylidene-1,2-oxazole-4-carbohydrazide (**MM5** derivative), containing an unsubstituted phenyl ring, has been considered a leading structure. Its immunosuppressive activity was moderate. Modifications of a leading structure may be achieved by exchanging substituents, increasing or decreasing the size of the ring, and simplifying or stiffening a molecule. The exchange of a substituent presents the simplest way to adjust a modified structure to an action (target) site. In the described **MM1**–**10** compound series, the location of a substituent in the phenyl ring was subject to modification. In these cases, the hydrogen atom was exchanged for a chlorine atom or a hydroxyl, metoxyl, or nitryl group. In addition, in the **MM4** compound, the phenyl ring (aromatic one) was exchanged for a heteroaromatic, 5-nitrothiophen-2-yl ring.

The assumption of these modifications was to obtain derivatives of a higher immunosuppressive activity in relation to lymphocyte proliferation and/or proinflammatory cytokine production and lower toxicity than the leading structure. The **MM5** derivative, containing an unsubstituted phenyl ring was characterized by a moderate suppression of lymphocyte proliferation. Literature data [37] indicate that the phenyl ring belongs to groups preferably binding to flat, hydrophobic sites by means of van der Waals bonds. The introduction of the hydroxyl group, which potentially belongs to groups interacting with an acting site by hydrogen bonds, increased the strength of the action of the **MM2** derivative in relation to the unsubstituted phenyl ring in the **MM5** leading structure. **MM2**, containing a 4-hydroxyphenyl group, exhibited higher suppressive potency than **MM5** in the described proliferation test. The most active in this series of compounds was 5-amino-*N*′-(2,4-dihydroxyphenyl)methylidene-*N*,3-dimethyl-1,2-oxazole-4-carbohydrazide (the **MM3** compound) containing a 2,4-dihydroxy phenyl substituent. A higher number of hydroxyl groups in **MM3** should in theory cause an increase of the molecule’s polarity and a possibility to form more hydrogen bonds in comparison with **MM2**, which contains only one hydroxyl group. An increased number of groups capable of forming hydrogen bonds lowers the susceptibility of a molecule to absorption, with the exception of compounds, such as methotrexate or erythromycin, which are transferred by transporting proteins [37]. 

It is known that derivatives containing nitroaromatic groups are metabolized to toxic compounds [37]. Our compounds, containing 4-nitrophenyl (**MM9**) or 3-nitrophenyl (**MM10**) fragments did not show toxicity in our test, but their suppressive actions were lower than that of **MM5**. Such a modification, based on the introduction of the nitryl group, worsened the biological activity but did not significantly influence toxicity. In turn, the exchange of the aromatic ring for the heteroaromatic one with the nitryl substituent led to a high increase in toxicity in the **MM4** compound. Literature reports [37] prove that derivatives containing 5-nitrophen-2-yl group display a higher pharmacological activity than derivatives bearing an unsubstituted aromatic ring. Bearing in mind that our compounds are expected to be potential immunosuppressive drugs in such immunological disorders, such as autoimmunity or inflammation, compound **MM4** was not attractive enough because of its toxicity with regard to the A549 reference cell line.

The exchange of the hydrogen atoms in the phenyl ring for electro-acceptor chlorine atoms in **MM6** and **MM7** compounds did not significantly increase their immunosuppressive potencies in comparison to **MM5**. These effects were observed in every concentration used. The replacement of the hydrogen atom by a big methoxyl group in the **MM8** derivative did not positively affect its activity in comparison with **MM5** leading structure. Thus, it may be concluded that both the lack of ability to form hydrogen bonds in **MM8**, in contrast with the **MM3** compound, as well as the introduction of the substituent to position 2 of the phenyl ring, may hinder adjustment of this fragment to a binding site.

The presented consequences of the structural modifications of the **MM1**–**10** compound series allow us to draw a conclusion that the possibility of forming hydrogenous bonds may have a major impact on the biological activity of the **MM3** compound displaying the highest activity. The interdependence between the structure, toxicity, and activity of the compounds is depicted in Table 1.

The mechanisms of the immunosuppressive actions of the isoxazole derivatives may differ. For example, leflunomide interferences with pyrimidine synthesis [36], and some isoxazoles may act as p38 mitogen-activated protein kinase (MAP kinase) inhibitors [38], or, as in the case of oxazolones, inhibit tyrosinase activity [39]. An isoxazole derivative, synthesized in our laboratory, MZO-2 (ethyl *N*-{4-[(2,4-dimethoxybenzyl)carbamoyl]-3-methylisoxazol-5-yl}acetimidate) [40], had no effect on the induction phase of the humoral immune response to sheep red blood cells (SRBC) in vitro and in vivo and moderately suppressed the induction phase of delayed-type hypersensitivity (DTH) to ovalbumin (OVA). Its inhibitory effect on carrageenan-induced paw inflammation was potent. Likewise, MZO-2 applied in ointment was very effective in reducing contact sensitivity to oxazolone compared with tacrolimus, the reference drug. Its mechanism of action we associated with inhibition of caspase 3, 8, and 9 expression in Jurkat cells, because caspases are essential for interleukin-2 (IL-2) release upon T cell activation [41]. In the case of 01 K compounds (4-phenyl-1-(5-amino-3-methylisoxazole-4-carbonyl)-thiosemicarbazide), suppressive in relation to interleukin-1β (IL-1β) and tumor necrosis factor α (TNF α) production by lipopolysaccharide (LPS)stimulated splenocytes and concanavalin A (ConA)–induced thymocyte proliferation [42], the compound upregulated in Jurkat cells’ expression of caspases 3 and 9, Fas and Bcl2, indicating induction of cell apoptosis as its major mechanism of action. The investigations on mechanism of action of **MM3** (Table 2) revealed some resemblance to the action of 01 K compounds indicating elicitation of cell apoptosis as a major cause of the immunosuppressive property of the compound. The results strongly suggest initiation by **MM3** of an apoptotic pathway associated with activation by caspase 8 of NFκB [43] and Fas [44]. On the other hand, based on the obtained data, the involvement of Bcl2 [45] and p53 [46] in this process seems to be excluded.

In conclusion, among the synthesized group of isoxazole derivatives, we selected the representative, nontoxic **MM3** compound with strong immunosuppressive properties and suggested its molecular mechanism of action. The compound is a good candidate for further studies in in vivo models to evaluate its potential therapeutic utility.

## 4. Materials and Methods

### 4.1. Chemistry

Melting points were determined by Büchi apparatus (Laboratoriums-Technik AG, Flawil, Switzerland) and Kofler system (Wagner & Munz) and were uncorrected. The progress of the reaction was monitored by thin layer chromatography (TLC) on silica gel Polygram SIL G/UV 254 nm coated TLC plates (Macherey-Nagel) and visualized by ultraviolet (UV) light at 254 nm (Fisher Bioblock Scientific 254 nm lamps). Infrared (IR) spectra were collected on a Thermo Scientific Nicolet iS50 FT-IR spectrometer with built-in iS50 ATR single reflection crystal. Frequencies are reported in cm^−1^. The samples were applied as solids. The proton nuclear magnetic resonance (^1^H NMR) spectra were obtained using a Bruker ARX 300 MHz NMR spectrometer in *d*_6_-dimethylsulfoxide (DMSO-*d*_6_). Chemical shifts are given in ppm units. Signal multiplicities are represented by the following abbreviations: s (singlet), d (doublet), t (triplet), and m (multiplet). Values of coupling constant are reported as *J* in Hz. Mass spectrometry (MS) was performed on a Bruker Daltonic Electrospray ionisation-Quadrupole-Time of Flight (ESI-Q-TOF) apparatus. Monoisotopic mass was calculated (calc.) by Compass Data Analysis 4.2. All chemicals were purchased from commercial suppliers. Dry solvents were obtained according to the standard procedure. 

*General Procedure for the Synthesis of N′-Substituted Derivatives of 5-Amino-*N*,3-dimethyl-1,2-oxazole-4-Carbohydrazide Compounds***MM1**–**MM10**. To 1 mmol of 5-amino-N,3-dimethyl-1,2-oxazole-4-carbohydrazide (obtained according to an analogously described method, patent PL 193939) dissolved in 10 mL of 2-propanol, 5 mL of relevant aldehyde and indium (III) trifluoromethanesulfonate were added. The mixture was stirred and heated at a boiling temperature (82 °C) for 4 h. At the end of the reaction (controlled in a TLC), the mixture was cooled. The solution was evaporated in vacuum from the mixture. The crude product, which separated out, was collected on a filter. The unrefined compound was purified by recrystallization in methanol. As a result, a pure product was obtained. 

*5-Amino-N′-(ethylidene)-N,3-dimethyl-1,2-oxazole-4-carbohydrazide* (**MM1**). *Anal.* C_8_H_12_N_4_O_2_ (m.w. 196.206 g/mol); m.p. 211–212 °C; yield 68%; FTIR (ATR, selected lines) *v*_max_/cm^−1^: 1605 (C=O), 3441 (NH_2_). ^1^H NMR (DMSO-*d*_6_) δ (ppm): 1.25 (s, 3H, CH_3_), 2.19 (s, 3H, CH_3_), 2.93 (s, 3H, CH_3_), 7.22 (s, 2H, NH_2_), 8.62 (s, 1H, CH). MS (ESI) [M + H]^+^ *m*/*z* 197.1054, calc. *m*/*z* 197.1033, [M + Na]^+^ *m*/*z* 219.0876, calc. *m*/*z* 219.0852.

*5-Amino-N′-(4-hydroxyphenyl)methylidene-N,3-dimethyl-1,2-oxazole-4-carbohydrazide* (**MM2**). *Anal.* C_13_H_14_N_4_O_3_ (m.w. 274.275 g/mol); m.p. 149–151 °C; yield 71%; FTIR (ATR, selected lines) *v*_max_/cm^−1^: 1615 (C=O), 3446 (NH_2_). ^1^H NMR (DMSO-*d*_6_) δ (ppm): 2.09 (s, 3H, CH_3_), 3.33 (s, 3H, CH_3_), 6.79–6.82 (d, *J* = 8.7 Hz, 2H, CH-aromat), 7.07 (s, 2H, NH_2_), 7.50–7.53 (d, *J* = 8.7 Hz, 2H, CH-aromat), 7.88 (s, 1H, CH), 9.807 (s, 1H, OH). MS (ESI) *m*/*z* [M + H]^+^ 275.1157, calc. *m*/*z* 275.1139, *m*/*z* [M + Na]^+^ 297.0976, calc. *m*/*z* 297.0958.

*5-amino-N′-(2,4-dihydroxyphenyl)methylidene-N,3-dimethyl-1,2-oxazole-4-carbohydrazide* (**MM3**). *Anal.* C_13_H_14_N_4_O_4_ (m.w. 290.275 g/mol); m.p. 211–212 °C; yield 73%; FTIR (ATR, selected lines) *v*_max_/cm^−1^: 1620 (C=O), 3442 (NH_2_). ^1^H NMR (DMSO-*d*_6_) δ (ppm): 2.08 (s, 3H, CH_3_), 3.33 (s, 3H, CH_3_), 6.28–6.32 (d, *J* = 8.6 Hz, 2H, CH-aromat), 7.11 (s, 2H, NH_2_), 7.36–7.39 (d, *J* = 8.6 Hz, 1H, CH-aromat), 8.05 (s, 1H, CH), 9.78 (s, 1H, OH), 10.25 (s, 1H, OH). MS (ESI) *m*/*z* [M + H]^+^ 291.1114, calc. *m*/*z* 291.1087, *m*/*z* [M + Na]^+^ 313.0930, calc. *m*/*z* 313.0907.

*5-Amino-N,3-dimethyl-N′-(5-nitrothiophen-2-yl)methylidene-1,2-oxazole-4-carbohydrazide* (**MM4**). *Anal.* C_11_H_11_N_5_O_4_S (m.w. 309.301 g/mol); m.p. 230–231 °C; yield 81%; FTIR (ATR, selected lines) *v*_max_/cm^−1^: 1628 (C=O), 3445 (NH_2_). ^1^H NMR (DMSO-*d*_6_) δ (ppm): 2.11 (s, 3H, CH_3_), 3.34 (s, 3H, CH_3_), 7.28 (s, 2H, NH_2_), 7.44–7.46 (d, *J* = 4.4 Hz, 1H, CH-aromat), 8.10–8.11 (d, *J* = 4.4 Hz, 1H, CH-aromat), 8.17 (s, 1H, CH). MS (ESI) *m*/*z* [M + H]^+^ 310.0630, calc. *m*/*z* 310.0604, *m*/*z* [M + Na]^+^ 332.0449, calc. *m*/*z* 332.0424.

*5-Amino-N,3-dimethyl-N′-phenylmethylidene-1,2-oxazole-4-carbohydrazide* (**MM5**). *Anal.* C_13_H_14_N_4_O_2_ (m.w. 258.276 g/mol); m.p. 134–135 °C; yield 75%; FTIR (ATR, selected lines) *v*_max_/cm^−1^: 1635 (C=O), 3443 (NH_2_). ^1^H NMR (DMSO-*d*_6_) δ (ppm): 2.09 (s, 3H, CH_3_), 3.37 (s, 3H, CH_3_), 7.13 (s, 2H, NH_2_), 7.40–7.43 (m, 3H, CH-aromat), 7.67–7.70 (d, *J* = 6.4 Hz, 2H, CH-aromat), 7.97 (s, 1H, CH). MS (ESI) *m*/*z* [M + H]^+^ 259.1215, calc. *m*/*z* 259.1189, *m*/*z* [M + Na]^+^ 281.1035, calc. *m*/*z* 281.1009.

*5-Amino-N′-(4-chlorophenyl)methylidene-N,3-dimethyl-1,2-oxazole-4-carbohydrazide* (**MM6**). *Anal.* C_13_H_13_N_4_O_2_Cl (m.w. 292.721 g/mol); m.p. 210–211 °C; yield 69%; FTIR (ATR, selected lines) *v*_max_/cm^−1^: 1628 (C=O), 3447 (NH_2_). ^1^H NMR (DMSO-*d*_6_) δ (ppm): 2.08 (s, 3H, CH_3_), 3.32 (s, 3H, CH_3_), 7.13 (s, 2H, NH_2_), 7.48–7.51 (d, *J* = 8.6 Hz, 2H, CH-aromat), 7.68–7.71 (d, *J* = 8.6 Hz, 2H, CH-aromat), 7.97 (s, 1H, CH). MS (ESI) *m*/*z* [M + H]^+^ 293.0811, calc. *m*/*z* 293.0800, *m*/*z* [M + Na]^+^ 315.0631, calc. *m*/*z* 315.0613.

*5-Amino-N′-(2-chlorophenyl)methylidene-N,3-dimethyl-1,2-oxazole-4-carbohydrazide* (**MM7**). *Anal.* C_13_H_13_N_4_O_2_Cl (m.w. 292.721 g/mol); m.p. 208–209 °C; yield 65%; FTIR (ATR, selected lines) *v*_max_/cm^−1^: 1630 (C=O), 3438 (NH_2_). ^1^H NMR (DMSO-*d*_6_) δ (ppm): 2.09 (s, 3H, CH_3_), 3.39 (s, 3H, CH_3_), 7.19 (s, 2H, NH_2_), 7.367–7.41 (m, 2H, CH-aromat), 7.51–7.52 (m, 1H, CH-aromat), 7.85–7.88 (m, 1H, CH-aromat), 8.06 (s, 1H, CH). MS (ESI) *m*/*z* [M + H]^+^ 293.0811, calc. *m*/*z* 293.0800, *m*/*z* [M + Na]^+^ 315.0631, calc. *m*/*z* 315.0619.

*5-Amino-N′-(2-methoxyphenyl)methylidene-N,3-dimethyl-1,2-oxazole-4-carbohydrazide* (**MM8**). *Anal.* C_14_H_16_N_4_O_3_ (m.w. 288.302 g/mol); m.p. 224–225 °C; yield 63%; FTIR (ATR, selected lines) *v*_max_/cm^−1^: 1625 (C=O), 3445 (NH_2_). ^1^H NMR (DMSO-*d*_6_) δ (ppm): 2.08 (s, 3H, CH_3_), 3.35 (s, 3H, CH_3_), 3.87 (s, 3H, OCH_3_), 6.95–7.09 (t, *J* = 8.9 Hz, 2H, CH-aromat), 7.12 (s, 1H, CH-aromat), 7.13 (s, 2H, NH_2_), 7.35–7.40 (m, 1H, CH-aromat), 8.08 (s, 1H, CH). MS (ESI) *m*/*z* [M + H]^+^ 289.1313, calc. *m*/*z* 289.1295, *m*/*z* [M + Na]^+^ 311.1132, calc. *m*/*z* 311.1115.

*5-Amino-N,3-dimethyl-N′-(4-nitrophenyl)methylidene-1,2-oxazole-4-carbohydrazide* (**MM9**). *Anal.* C_13_H_13_N_5_O_4_ (m.w. 303.273 g/mol); m.p. 214–216 °C; yield 73%; FTIR (ATR, selected lines) *v*_max_/cm^−1^: 1635 (C=O), 3446 (NH_2_). ^1^H NMR (DMSO-*d*_6_) δ (ppm): 2.09 (s, 3H, CH_3_), 3.39 (s, 3H, CH_3_), 7.20 (s, 2H, NH_2_), 7.89–7.92 (d, *J* = 8.9 Hz, 2H, CH-aromat), 8.08 (s, 1H, CH), 8.27–8.31 (d, *J* = 8.9 Hz, 2H, CH-aromat). MS (ESI) *m*/*z* [M + H]^+^ 304,1055, calc. *m*/*z* 304.1040, *m*/*z* [M + Na]^+^ 326.0875, calc. *m*/*z* 326.0860.

*5-Amino-N,3-dimethyl-N′-(3-nitrophenyl)methylidene-1,2-oxazole-4-carbohydrazide* (**MM10**). *Anal.* C_13_H_13_N_5_O_4_ (m.w. 303.273 g/mol); m.p. 209–210 °C; yield 77%; FTIR (ATR, selected lines) *v*_max_/cm^−1^: 1638 (C=O), 3438 (NH_2_). ^1^H NMR (DMSO-*d*_6_) δ (ppm): 2.10 (s, 3H, CH_3_), 3.34 (s, 3H, CH_3_), 7.19 (s, 2H, NH_2_), 7.69–7.75 (t, *J* = 8.1 Hz, 1H, CH-aromat), 8.08–8.12 (d, *J* = 8.1 Hz, 2H, CH-aromat), 8.19–8.23 (m, 1H, CH-aromat), 8.53 (s, 1H, CH). MS (ESI) *m*/*z* [M + H]^+^ 304.1059, calc. *m*/*z* 304.1040, *m*/*z* [M + Na]^+^ 326.0876, calc. *m*/*z* 326.0860.

Additional spectroscopic data is available in the Supplementary Materials (Figures S4–S21). 

### 4.2. Biology

#### 4.2.1. Reagents

Fetal calf serum (FCS), RPMI-1640, and Hanks’ medium were purchased from Biowest (Nuaillé, France). Lipopolysaccharide from *Escherichia coli* 0111:B4 (LPS), phytohemagglutinin (PHA), dimethyl sulfoxide (DMSO), and MTT (3-[4,5-dimethylthiazol-2-yl]-2,5-diphenyltetrazolium bromide) and all other reagents were from Sigma-Aldrich (St. Louis, MO, USA). 

#### 4.2.2. Preparation of the Compounds for Biological Assays

The compounds were dissolved in DMSO to a concentration of 10 mM and kept at 4 °C. Then, the compound solutions were incubated at 37 °C for 10 min with vigorous shaking and subsequently diluted in the culture medium to the respective concentrations used in the cell cultures.

#### 4.2.3. Determination of the Toxicity of the MM Compounds against the A549 Cell Line 

The cytotoxicity of the compounds was determined by measuring growth of human lung epithelial A549 cells (ATCC CCL 185). The cells were suspended in a density of 2 × 10^6^ mL in a culture medium, referred to below as “the culture medium”, consisting of RPMI-1640 with the addition of 10% fetal calf serum (FCS), 100 U/mL of penicillin, 100 μg/mL streptomycin, and 2 mM L-glutamine. The compounds were tested at a concentration range of 100–12.5 µM and the **MM3** compound at 250–1.9 µM. The test was performed in 96-flat bottom plates containing 2 × 10^5^ cells incubated initially for 24 h in a cell culture incubator. Then, the supernatants were removed and to the cells, appropriate dilutions of the compounds (0.2 mL) were added in a cell culture medium containing 2% FCS. The cultures were incubated for 72 h followed by the determination of cell viability by a colorimetric MTT method. In parallel, control cultures containing appropriate dilutions of the solvent (DMSO) were also incubated. The results were presented as mean optical density (OD) values from four wells ± standard deviation.

#### 4.2.4. Isolation of the Peripheral Blood Mononuclear Cells (PBMCs)

Venous blood from a single donor was withdrawn into heparinized syringes and diluted twice with phosphate buffered saline (PBS). PBMCs were isolated by centrifugation on a Ficoll-Uropoline gradient (density 1.077 g/mL) at 800× *g* for 20 min at 4 °C. The interphase cells were then washed three times with Hanks’ medium and re-suspended in the culture medium at a density of 2 × 10^6^ cells/mL.

#### 4.2.5. PHA-Induced Proliferation of Human PBMC

PBMC were distributed into 96-well flat-bottom plates in 100 µL aliquots (2 × 10^5^ cells/well). PHA was added at a concentration of 5 µg/mL. The compounds were tested at the following doses: 100, 50, and 25 uM. DMSO at appropriate dilutions served as the control. After three days of incubation in a cell culture incubator, the proliferative response of the cells was determined by colorimetric MTT [47]. The results were presented as optical density (OD) values, and appropriate DMSO dilutions served as the control.

#### 4.2.6. Lipopolysaccharide-Induced TNF-a Production in Whole Blood Cell Culture

Human whole blood was diluted 10× with RPMI-1640 medium and distributed to 24-well culture plates in 1 mL aliquots. The cultures were stimulated with LPS (100 ng/mL), and the studied compound was added at concentrations of 50, 25, 12.5, and 6.15 µM. The control cultures contained DMSO in appropriate concentrations. After an overnight incubation, the supernatants were harvested and frozen at −80 °C until cytokine determination. TNF α concentrations were determined in the supernatants by using an ELISA kit from eBioscience in a presence of TNF α standard and originally expressed in pg/mL. 

#### 4.2.7. Colorimetric MTT Assay for Cell Growth and Kill

The assay was performed according to [47]. Briefly, 25 µL of MTT (5 mg/mL) stock solution was added per well at the end of cell incubation period, and the plates were incubated for an additional 3 h in a cell culture incubator. Then, 100 µL of the extraction buffer (20% SDS with 50% dimethylformamide (DMF), pH 4.7) was added. After an overnight incubation, the OD was measured at 550 nm with the reference wavelength of 630 nm in a Dynatech 5000 spectrophotometer. 

#### 4.2.8. Cultures of Jurkat Cells and Total RNA Isolation

Jurkat cells (10^5^/mL) in the culture medium were cultured overnight with the **MM3** compound (50 μM) Total RNA isolation was carried with TRIzol Reagent (Ambion) according to the manufacturer’s recommendations. The cell pellet (2 × 10^6^ cells) was suspended in 1 mL of TRIzol reagent, shaken, incubated for 10 min at room temperature (RT), supplemented with 0.2 mL of chloroform, shaken vigorously for 15 s, incubated for 3 min at RT, and centrifuged at 12,000× *g* for 15 min at 4 °C. The water phase was collected, transferred to a new tube, supplemented with 0.5 mL of isopropanol, incubated at RT for 10 min, and centrifuged at 12,000× *g* for 10 min at 4 °C. The RNA pellet was washed with 1 mL of 75% ethanol, dried in air, and dissolved in 20–30 μL of sterile diethylpyrocarbonate-treated Mili-Q water. RNA samples were stored at −20 °C. 

#### 4.2.9. Reverse Transcription

Single stranded complementary DNA (cDNA) was synthesized with oligo (dT) 12–18 primers from 5 μg of total RNA using Novazym VerteKit, according to the manufacturer’s instruction.

#### 4.2.10. Quantitation of Gene Expression by Real Time PCR

The expression of the genes (i.e., caspase 3, 7, 8, and 9, Bcl-2, Fas, NFκB1, and p53) were measured using an APA SYBR FAST qPCR Kit. The sequences of primers are enclosed in the Supplementary Materials. The reaction was performed in an Applied Biosystems ViiA7 thermocycler starting with 5 min of preincubation at 95 °C, followed by 35 amplification cycles as follows: 95 °C for 30 s and simultaneous annealing-extension-data acquisition for 45 s and 60 °C. Glyceraldehyde-3-phosphate dehydrogenase (GAPDH) was used as a housekeeping gene for arbitrary unit calculation for every tested gene.

#### 4.2.11. Statistics

The results are presented as mean values ± standard deviation (SD). The Brown–Forsythe test was used to determine the homogeneity of the variance between the groups. When the variance was homogenous, analysis of the variance (one-way ANOVA) was applied, followed by post hoc comparisons with the Tukey test to estimate the significance of the difference between the groups. Nonparametric data were evaluated with the Kruskal–Wallis analysis of variance. The significance was determined at *p* < 0.05. Statistical analysis was performed using STATISTICA 6.1 for Windows.

## Figures and Tables

**Table 1:** Interdependence between structure, toxicity, and biological activities of the studied compounds.

Compound	Modification/R 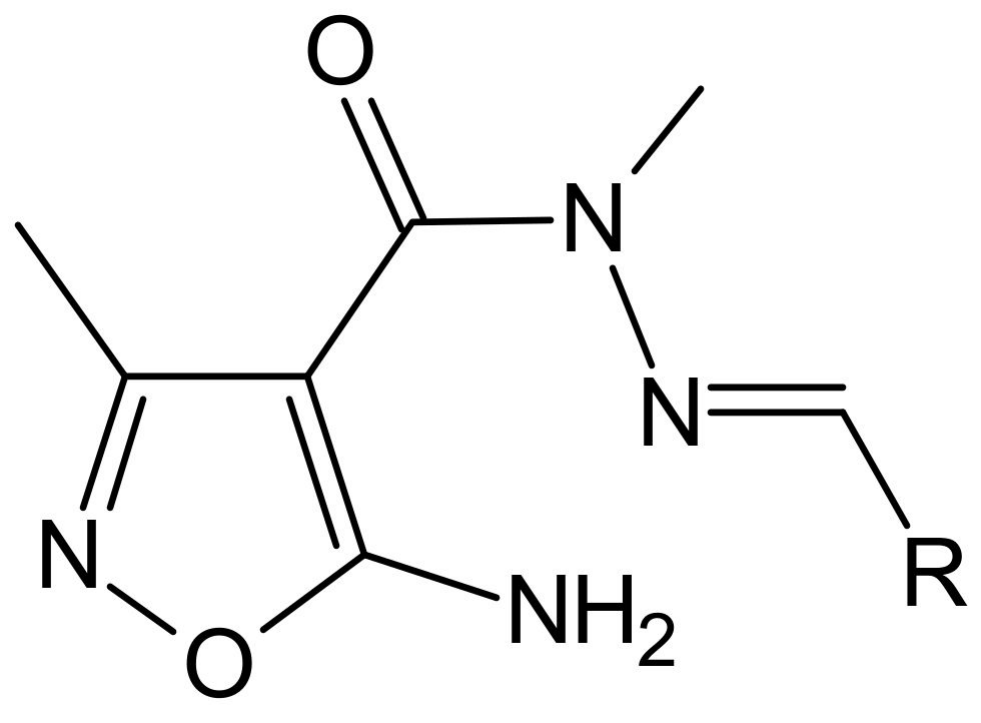	Toxicity against A549 Cells	Biological Activity
**MM1**	ethylidene	absent	not suppressive in the proliferation test
**MM2**	4-hydroxyphenyl	absent	moderately suppressive in the proliferation test
**MM3**	2,4-dihydroxyphenyl	absent	suppression of PBMC proliferation, TNF α activity and induction of a proapoptotic pathway
**MM4**	5-nitrothiophen-2-yl	high	strongly suppressive in the proliferation test
**MM5**	phenyl	absent	not suppressive in the proliferation test
**MM6**	4-chlorophenyl	moderate	moderately suppressive in the proliferation test
**MM7**	2-chlorophenyl	absent	weakly suppressive in the proliferation test
**MM8**	2-methoxyphenyl	absent	weakly suppressive in the proliferation test
**MM9**	4-nitrophenyl	moderate	moderately suppressive in the proliferation test
**MM10**	3-nitrophenyl	absent	moderately suppressive in the proliferation test

**Table 2:** The changes in expression of selected signaling molecules induced by **MM3** in cultures of Jurkat cells. Glyceraldehyde-3-phosphate dehydrogenase (GAPDH) was used as a housekeeping gene to normalize values obtained for the target genes. The values presented in the table reflect relative fold changes in response of the cells to the **MM3** compound, when the GAPDH gene expression was a control and assumed as equal to one.

Signaling Molecule	MM3 (50 μM)
Bcl-2	**0.5**
Casp-3	**20.6**
Casp-7	**16.0**
Casp-8	**50.7**
Casp-9	**2.7**
Fas	**7.8**
NF-κB1	**50.1**
p53	**0**

## Supplementary Materials

Supplementary materials includes detailed experimental procedures, characterizations of all new compounds, as well as additional figures and tables.
