# Supplementary material for: Synthesis, Immunosuppressive Properties, and Mechanism of Action of a New Isoxazole Derivative
Source: Molecules. 2018 Jun 26;23(7):1545. doi: 10.3390/molecules23071545 (PMC6099534; doi:10.3390/molecules23071545)

This report was created by ACD/NMR Processor Academic Edition. For more information go to www.acdlabs.com/nmrproc/

Figure S13. 1HNMR spectrum of compound MM2

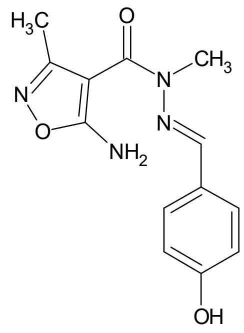

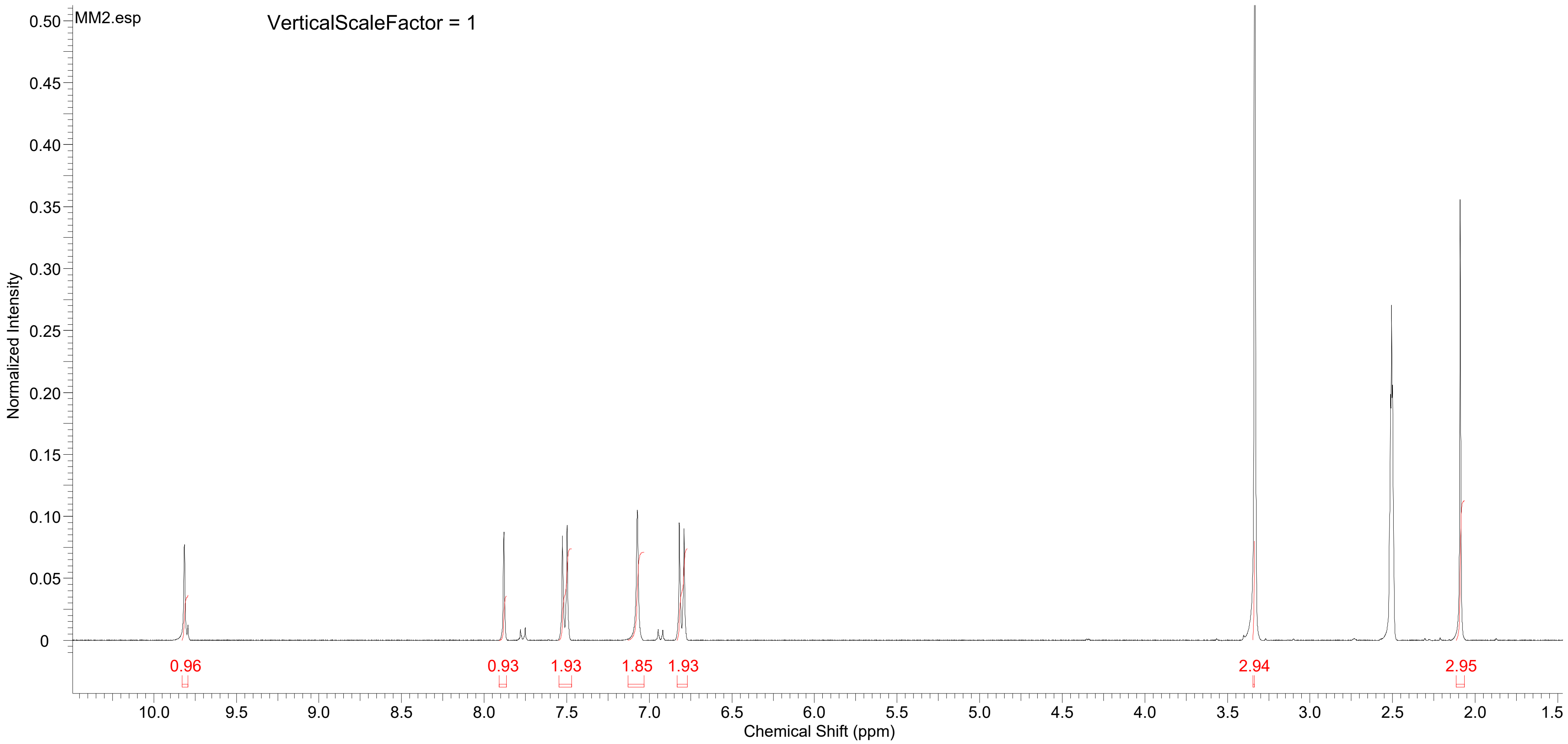

This report was created by ACD/NMR Processor Academic Edition. For more information go to www.acdlabs.com/nmrproc/

Figure S14. 1HNMR spectrum of compound MM3

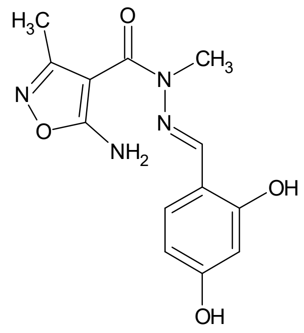

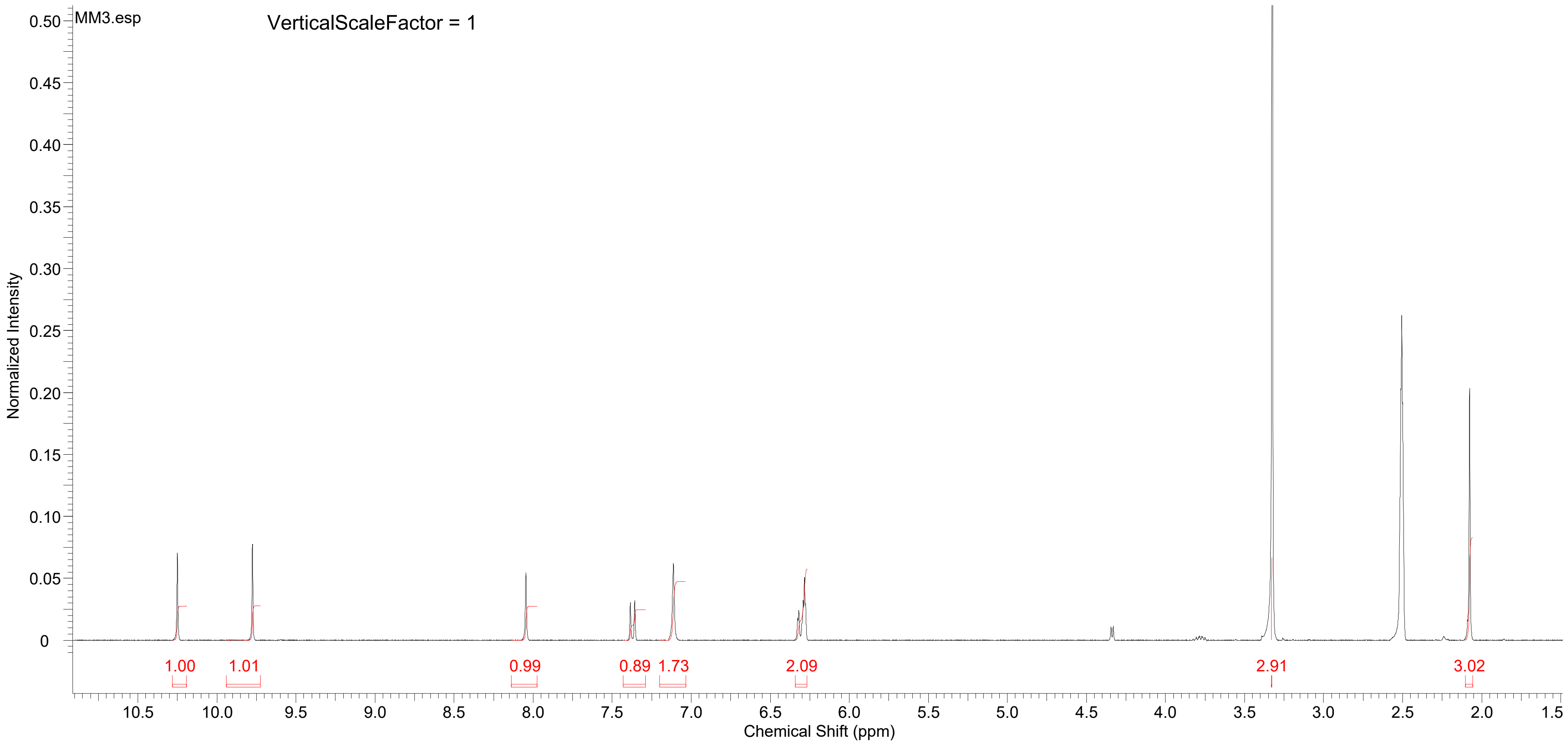

This report was created by ACD/NMR Processor Academic Edition. For more information go to www.acdlabs.com/nmrproc/

Figure S15. 1HNMR spectrum of compound MM4

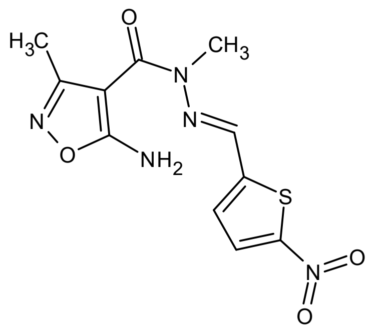

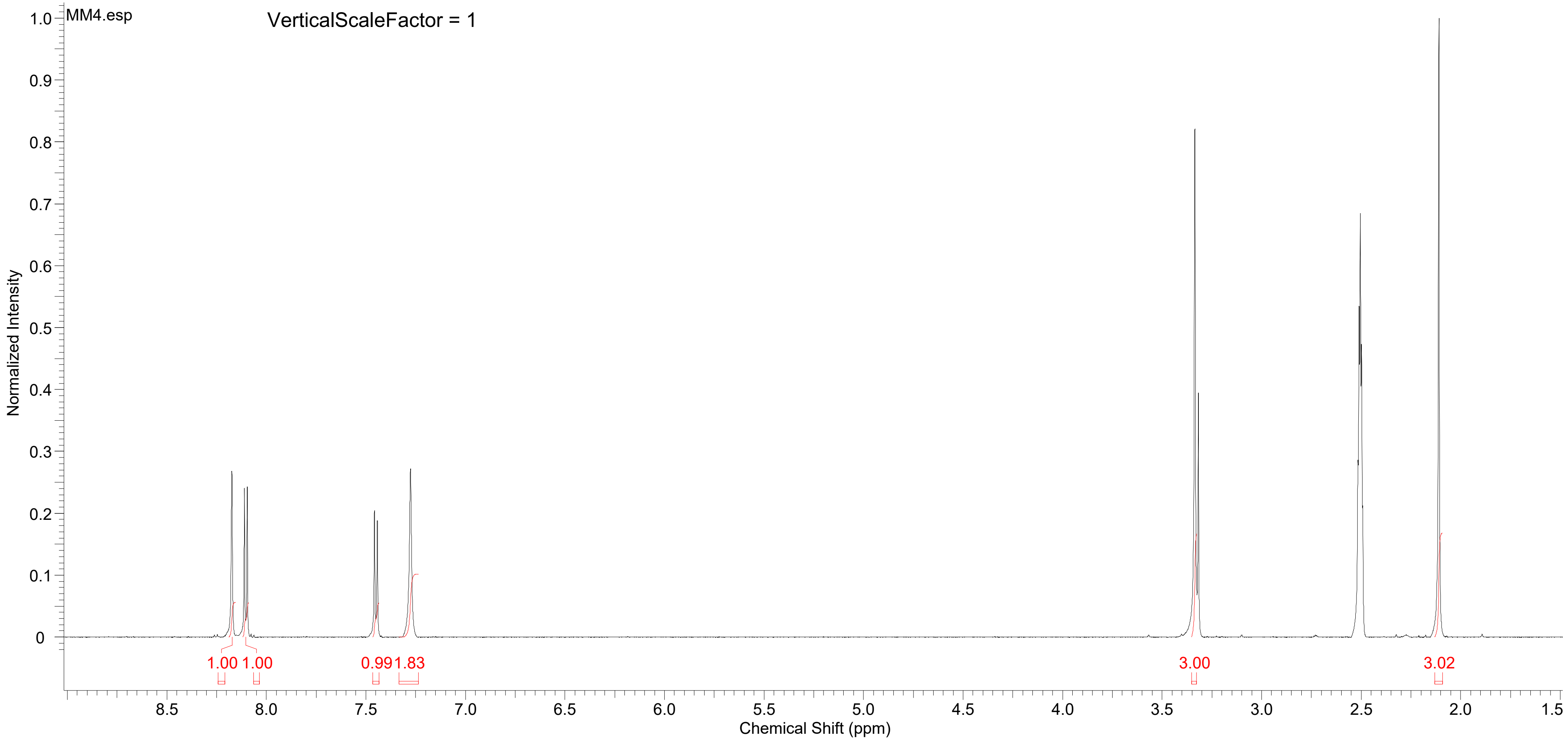

This report was created by ACD/NMR Processor Academic Edition. For more information go to www.acdlabs.com/nmrproc/

Figure S16. 1HNMR spectrum of compound MM5

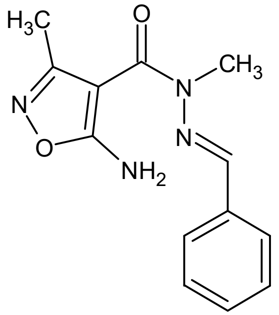

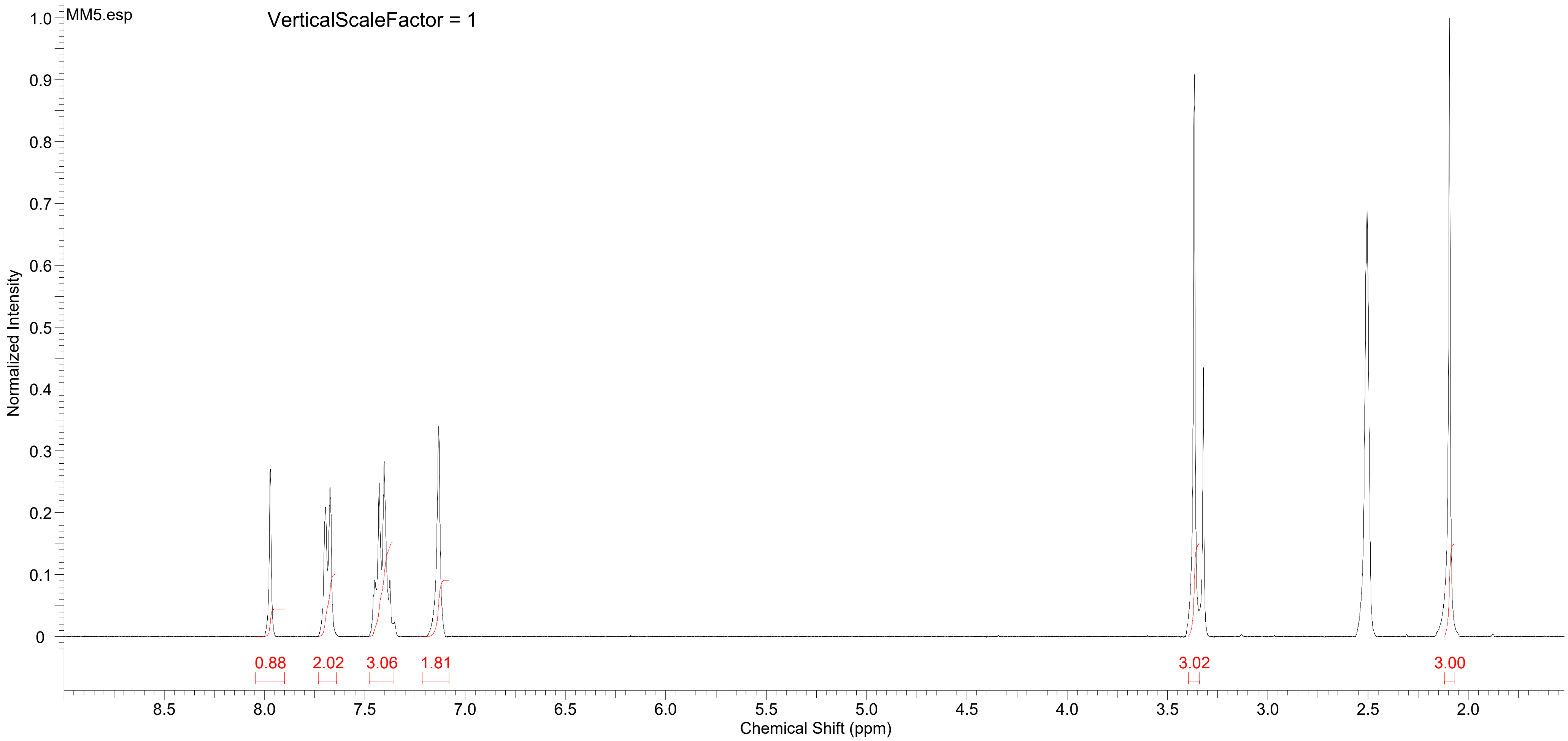

This report was created by ACD/NMR Processor Academic Edition. For more information go to www.acdlabs.com/nmrproc/

Figure S17. 1HNMR spectrum of compound MM6

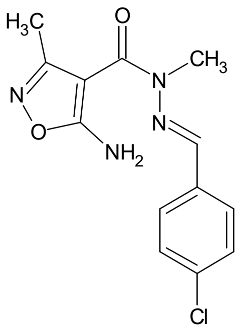

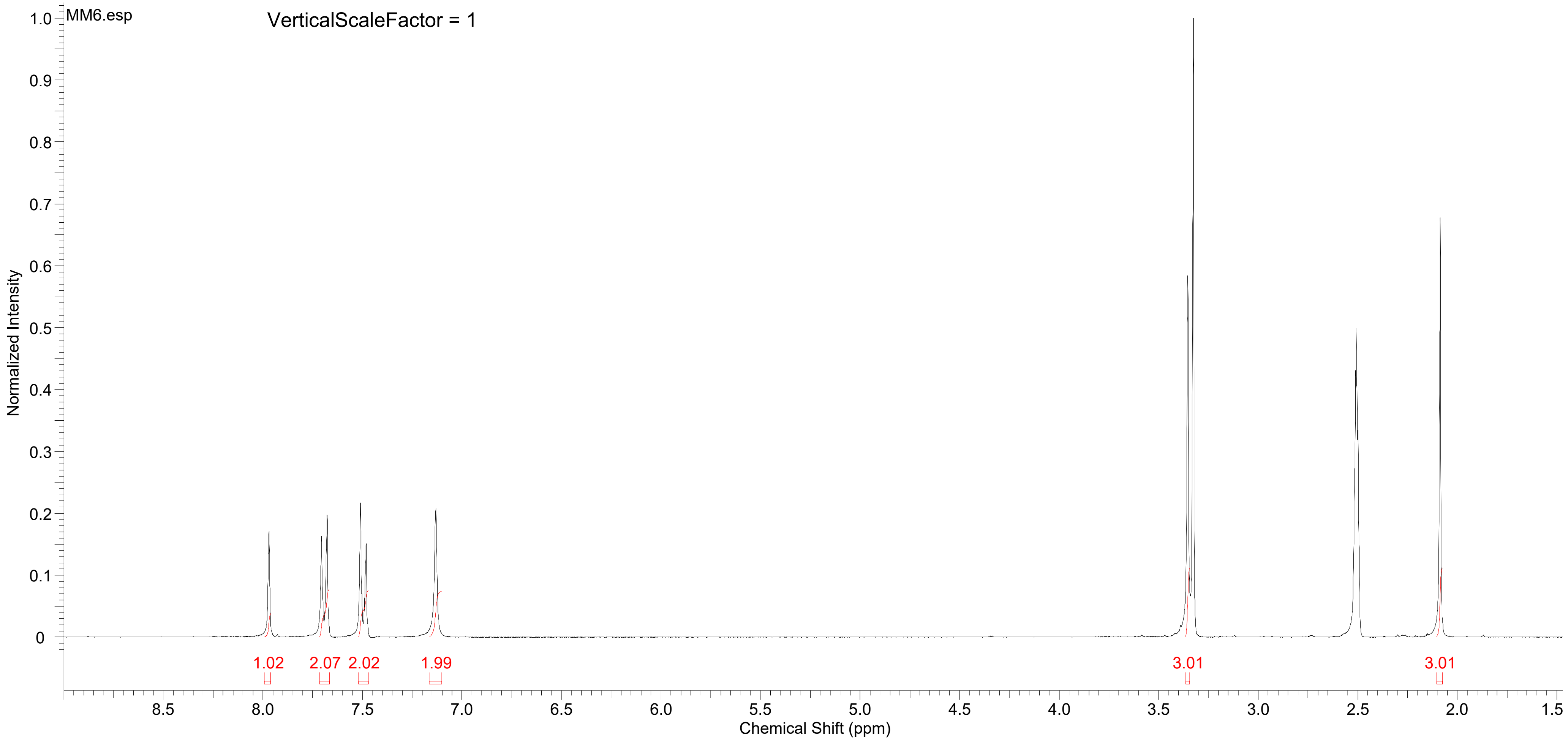

This report was created by ACD/NMR Processor Academic Edition. For more information go to www.acdlabs.com/nmrproc/

Figure S18. 1HNMR spectrum of compound MM7

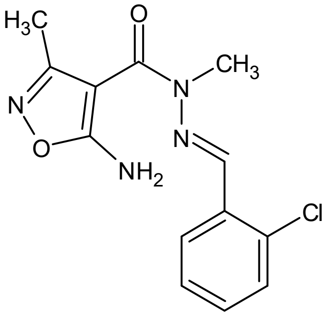

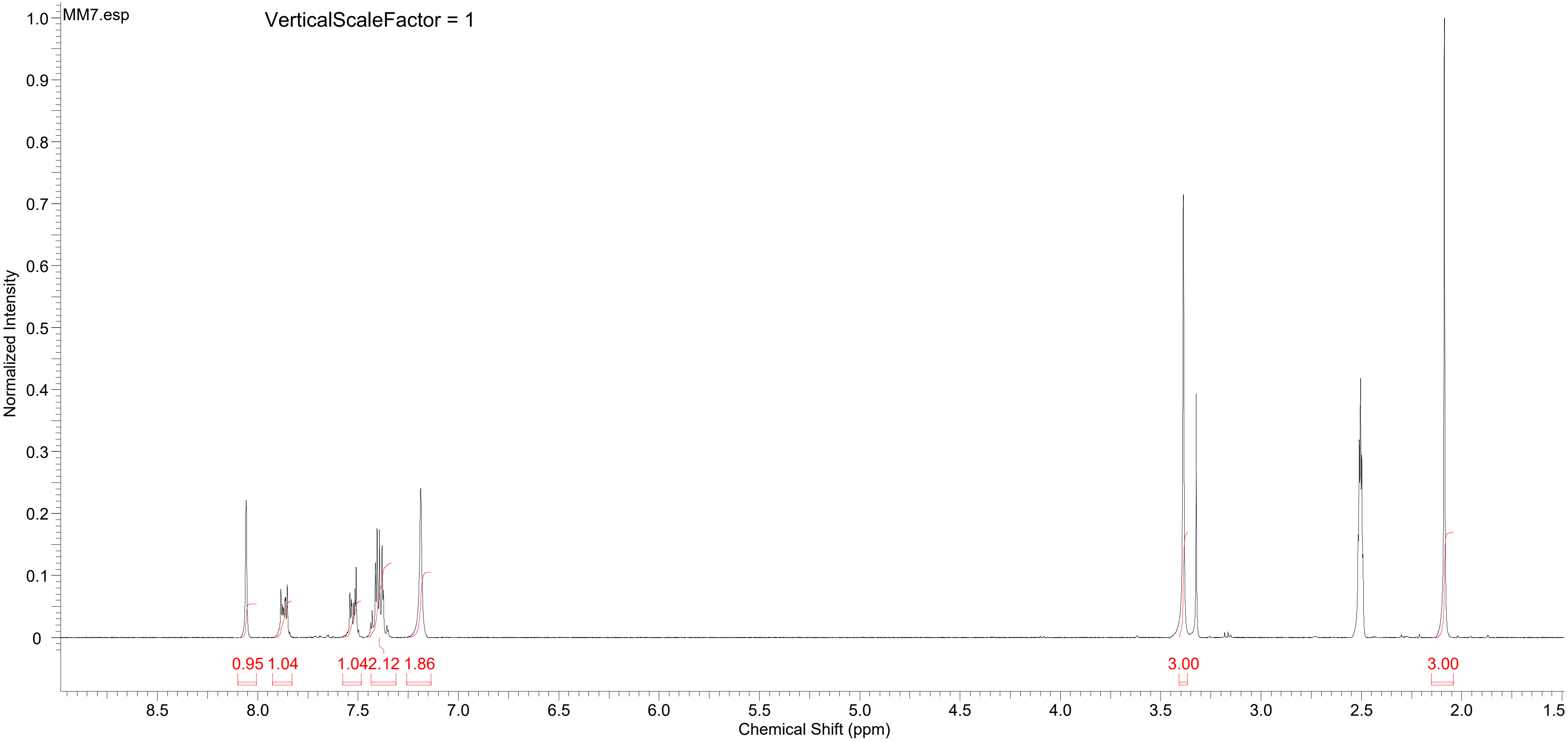

This report was created by ACD/NMR Processor Academic Edition. For more information go to www.acdlabs.com/nmrproc/

Figure S19. 1HNMR spectrum of compound MM8

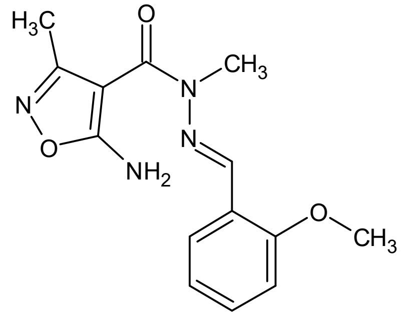

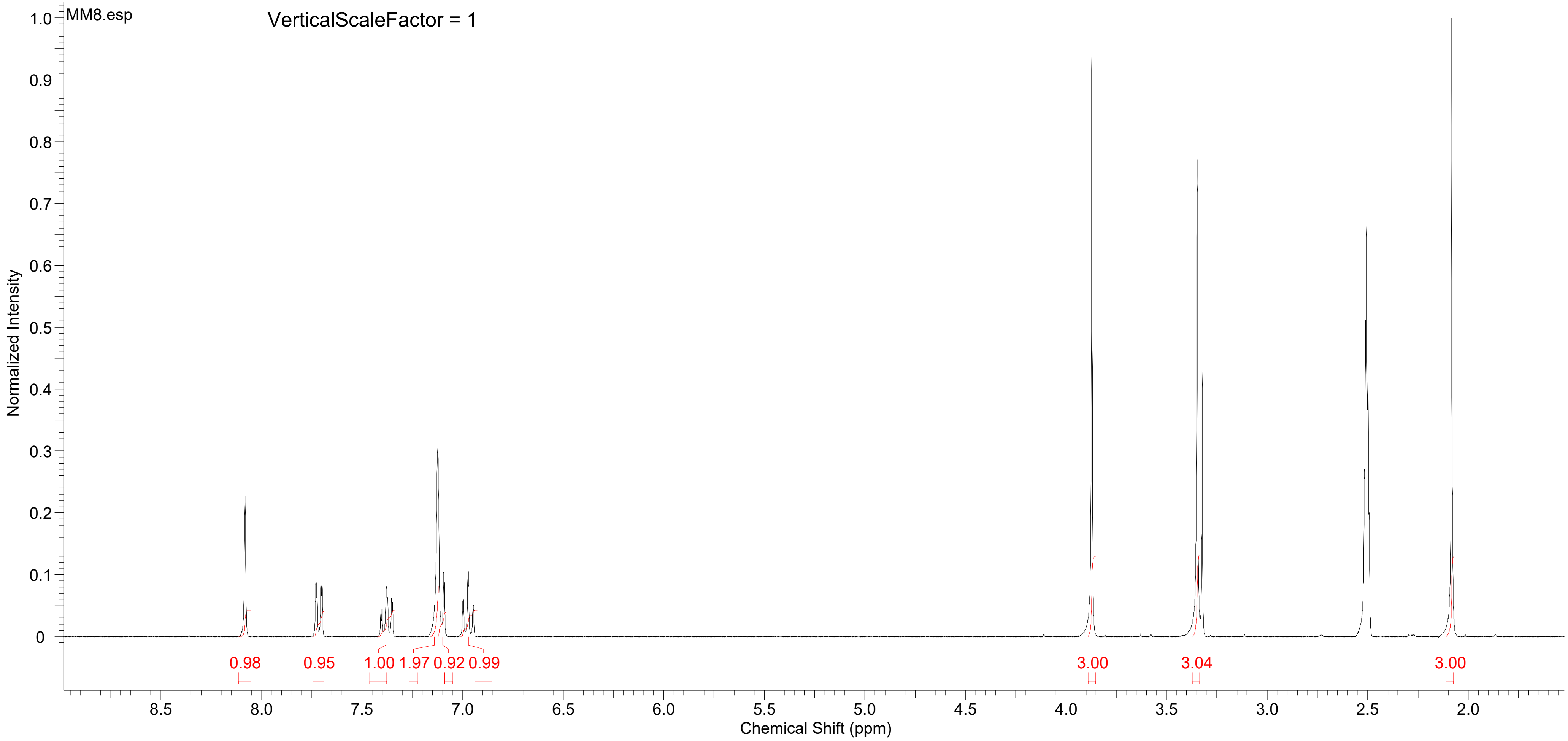

This report was created by ACD/NMR Processor Academic Edition. For more information go to www.acdlabs.com/nmrproc/

Figure S20. 1HNMR spectrum of compound MM9

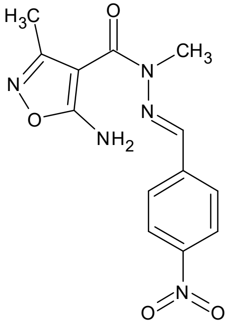

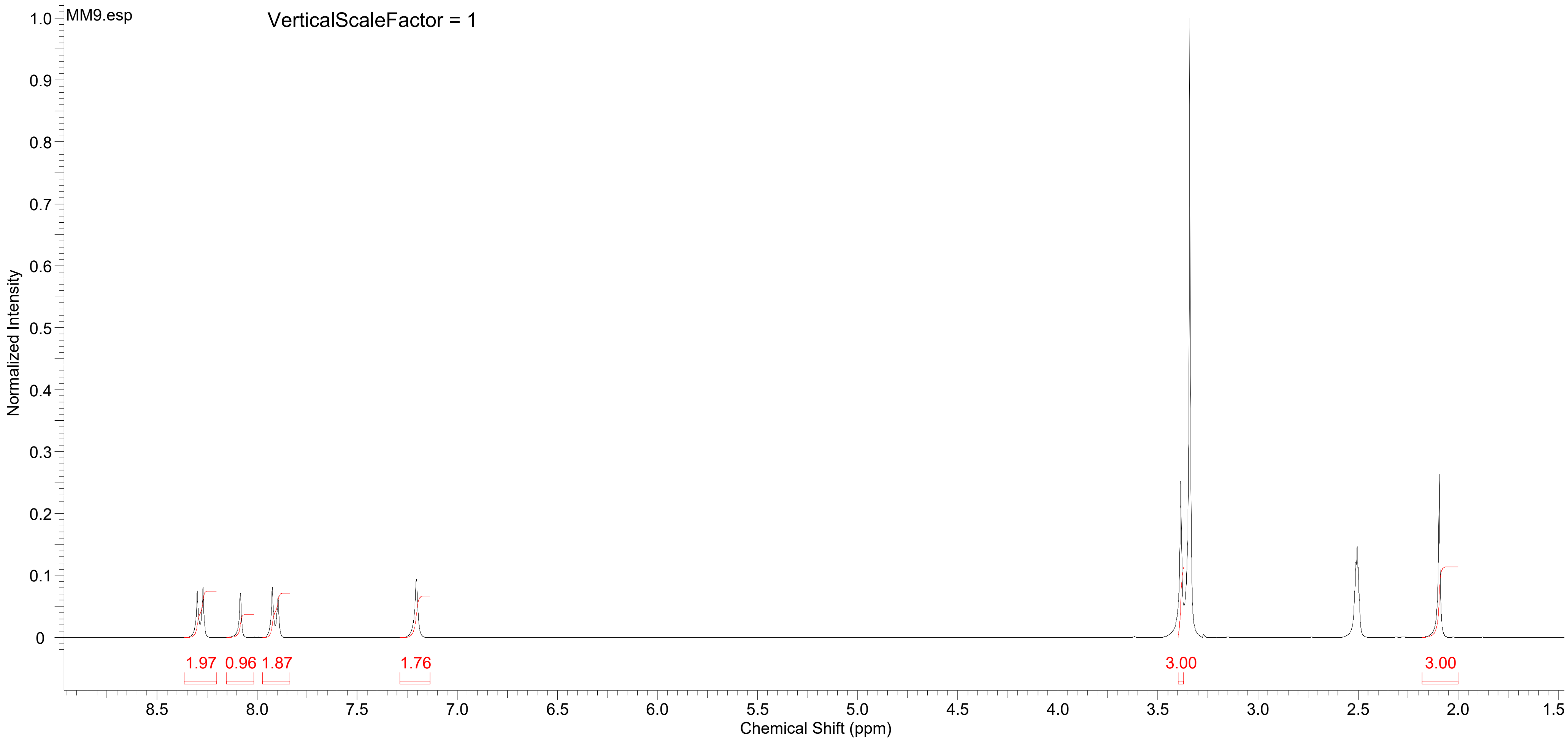

This report was created by ACD/NMR Processor Academic Edition. For more information go to www.acdlabs.com/nmrproc/

Figure S21. 1HNMR spectrum of compound MM10

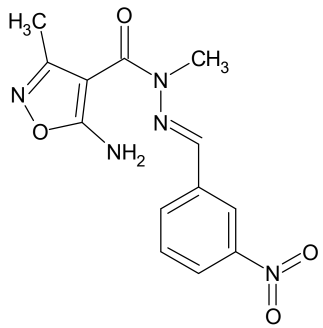

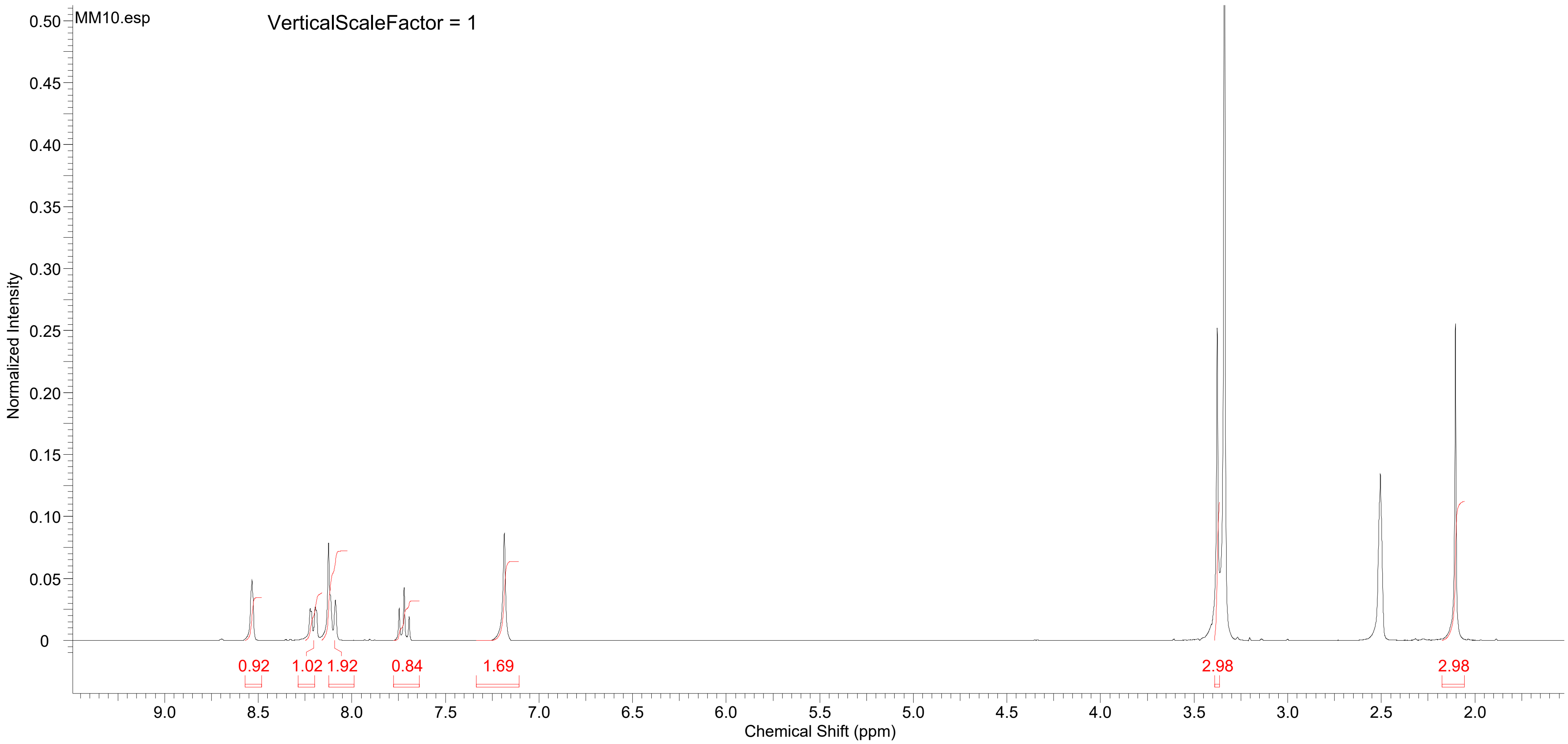

Supplement: Supplementary file 1 [file molecules-23-01545-s001.zip › molecules-321820-Sup-to publish/HNMR do supplementary.pdf]
