# Supplementary material for: Synthesis, Immunosuppressive Properties, and Mechanism of Action of a New Isoxazole Derivative
Source: Molecules. 2018 Jun 26;23(7):1545. doi: 10.3390/molecules23071545 (PMC6099534; doi:10.3390/molecules23071545)

Supplementary Material

Methods

Preparation of the Compounds for the Experiments

The working concentrations of the compounds were prepared from a stock solution (10 mM dissolved in DMSO) and stored at 4° C . The stock solution was incubated at 37° C for 10 min with vigorous shaking before further dilution in the culture medium.

Evaluation of Toxicity

L929 cells: The investigation was performed with L929 fibrosarcoma cells (American Type Culture Collection, Cell Culture Line - ATCC CCL 1). L929 cells were cultured in Eagle's (MEM) medium with addition of 10% inactivated FCS and 100 U/ml penicillin, 100 μg/ml streptomycin and 2 mM L-glutamine. The cells were cultured for 24h in 96-flat-bottom microtiter tissue culture plates in density of 2 x 10^6/mL. After the incubation the supernatants were removed and appropriate concentrations of the compounds (100 - 12.5 μM) diluted in the culture medium in a volume of 200 μL were added to the monolayer of L929 cells, followed by 72h incubation in a cell culture incubator. Cultures containing respective dilutions of DMSO served as control. After the incubation the cell viability was determined using MTT colorimetric method [1].
A549 cells: The investigation was carried out with A549 – tumor epithelial lung cells (ATCC CCL 185). A549 cells were cultured in RPMI 1640 medium with addition of 10% FCS, 100 U/ml penicillin, 100 μg/ml streptomycin and 2 mM L-glutamine. The compounds were tested at a concentration range of 250 – 12.5 μM. The cells were distributed to 96-well culture plates at density of 2 x 10^5/well and appropriate concentrations of the compounds, diluted in the culture medium containing 2% FCS, were added. After 72h incubation the cell viability was determined using the colorimetric MTT method [1].

Proliferation Test

The test was performed using spleenocytes from C57Bl/6 mice. The cells, suspended in the culture medium, were distributed to 96-well plates (2 x 10^5/well). The compounds were used at a 75 - 12.5 μM. concentration range. Appropriate DMSO dilutions served as control cultures. Leflunomide in DMSO solution and cyclosporine A (CSA) (Sigma-Aldrich) served as reference compounds. After 72h incubation the rate of cell proliferation was determined by MTT method.

Statistics

The results are presented as mean values ± standard deviation (SD). Brown-Forsyth's test was used to determine the homogeneity of variance between groups. When the variance was homogenous, analysis of variance (one-way ANOVA) was applied, followed by post hoc comparisons with the Tukey's test to estimate the significance of the difference between groups. Nonparametric data were evaluated with the Kruskal-Wallis' analysis of variance. Significance was determined at $p < 0.05$. Statistical analysis was performed using STATISTICA 6.1 for Windows.

Results

Evaluation of Cell Toxicity

MM compounds were tested for their potential toxicity with regard to fibrosarcoma L929 up to a concentration of 100 μM) and epithelial lung A549 cell lines (up to 250 μM). Control cultures contained DMSO at respective concentrations in the culture medium. The results (Figure S1 and S2) showed that in these experimental conditions the compounds were not toxic against the applied cell lines.

Figure S1. Toxicity of MM1-10 compounds with regard to L929 cells. Cytotoxicity of the compounds was determined by measuring growth of mouse L929 cells. The results are presented as mean optical density (OD) values from four wells ± standard deviation.

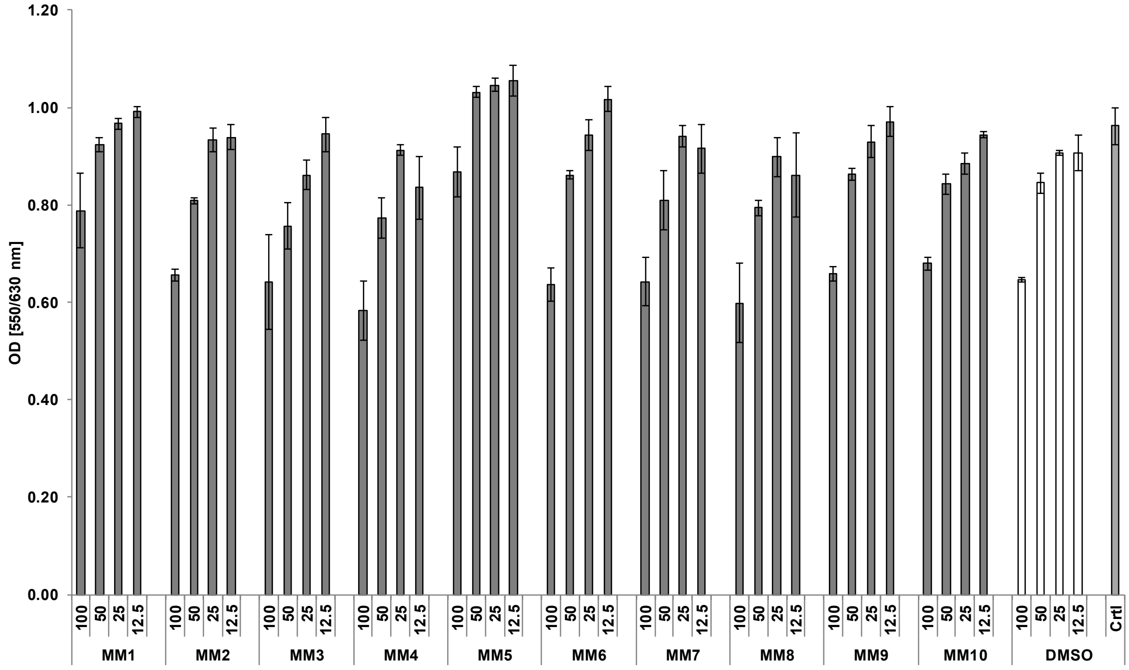

Figure S2. Toxicity of MM3 compounds against A549 cells. Cytotoxicity of the compounds was determined by measuring growth of human A549 cell in MTT colorimetric assay [1]. Appropriate dilutions of the solvent (DMSO) served as control cultures. The results are presented as mean optical density (OD) values from four wells ± standard deviation. Statistics: *, $p < 0.05$

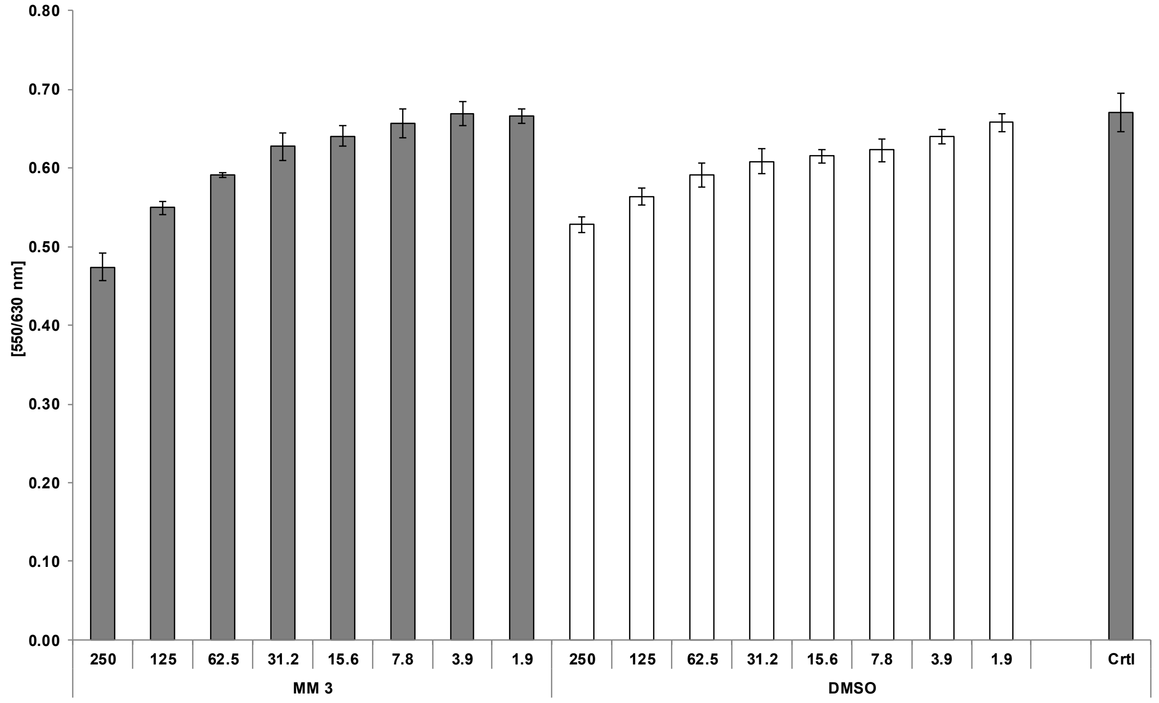

The effects of the compounds on concanavalin A (Con A) –induced splenocyte proliferation

The results (Figure S3) showed that the effects of the compounds on the mitogen-induced cell proliferation were differential and dose-dependent. The suppressive effects were none or negligible (MM1, MM5, MM9 and MM10) but strong in the case of MM3 and MM2. MM4 and MM8 compounds were not tested in this model.

Figure S3. The effects of MM1-10 compounds on concanavalin A induced splenocyte proliferation. The results are presented as mean optical density (OD) values from four wells ± standard deviation. Statistics: *, $p < 0.05$ versus appropriate dilutions of the solvent (DMSO).

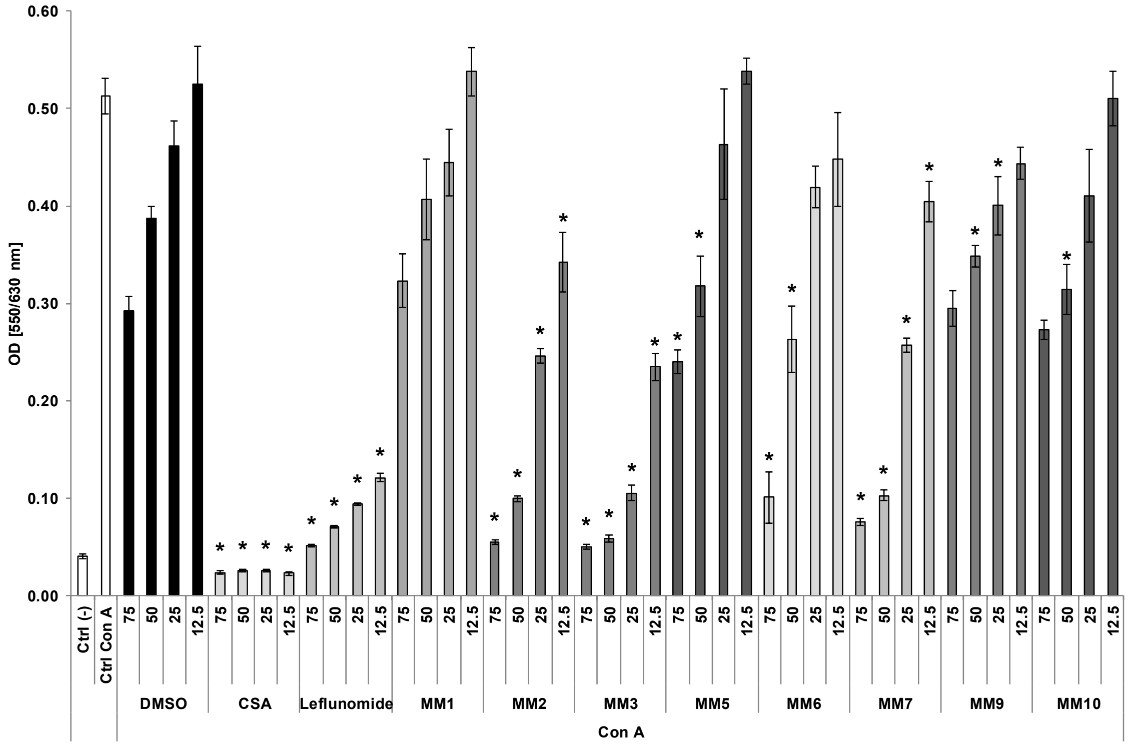

References

1. Hansen, M. B.; Nielsen, S. E.; Berg, K. Re-examination and further development of a precise and rapid dye method for measuring cell growth/cell kill. *J. Immunol. Methods* **1989**, *119*, 203–210;

Figure S4. ESI-MS spectrum of compound MM1.

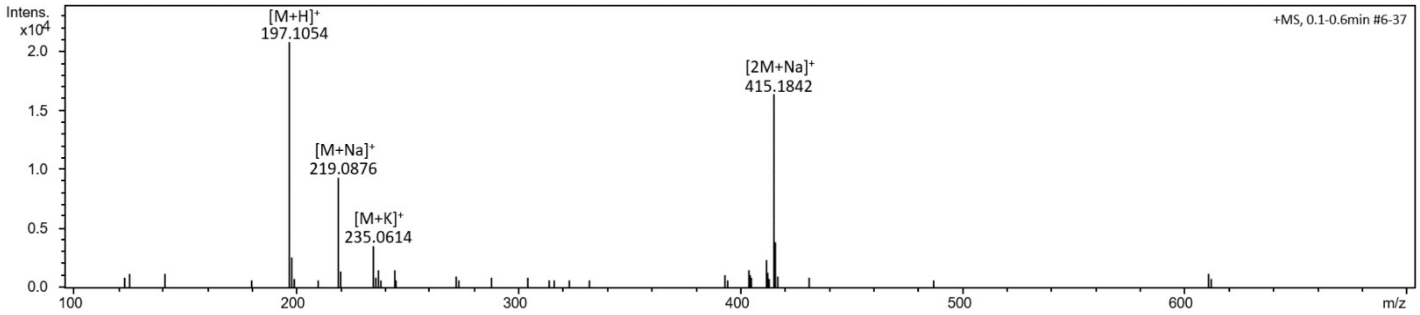

Figure S5. ESI-MS spectrum of compound MM2.

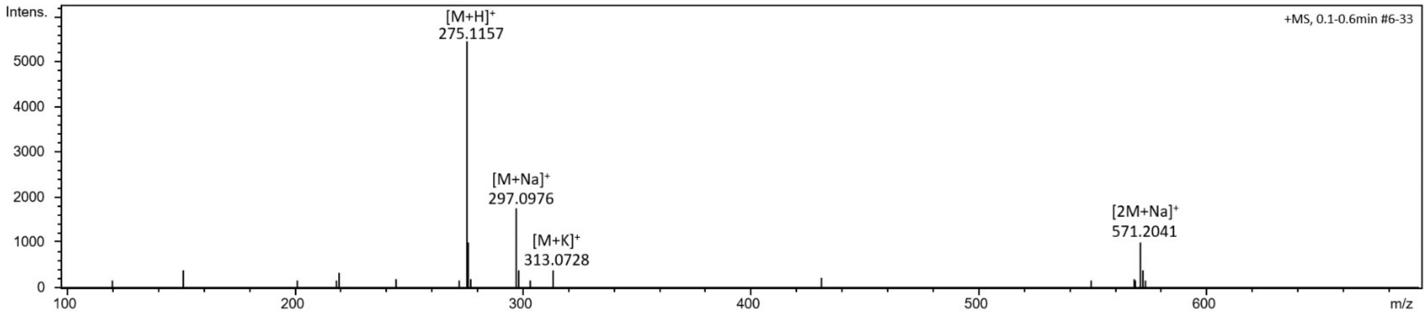

Figure S6. ESI-MS spectrum of compound MM3.

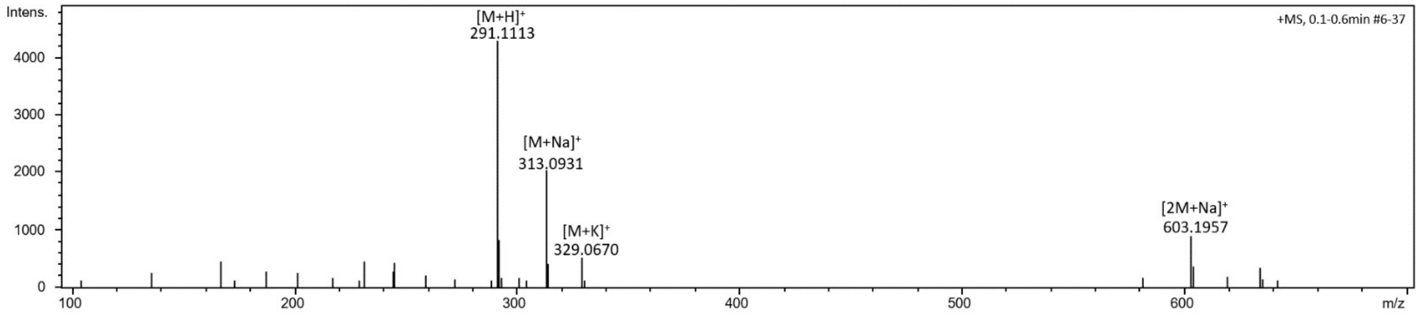

Figure S7. ESI-MS spectrum of compound MM5.

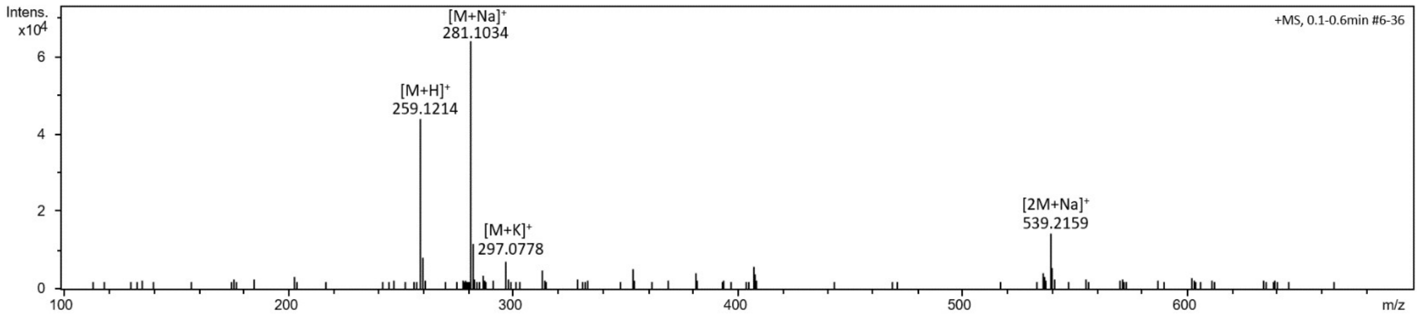

Figure S8. ESI-MS spectrum of compound MM6.

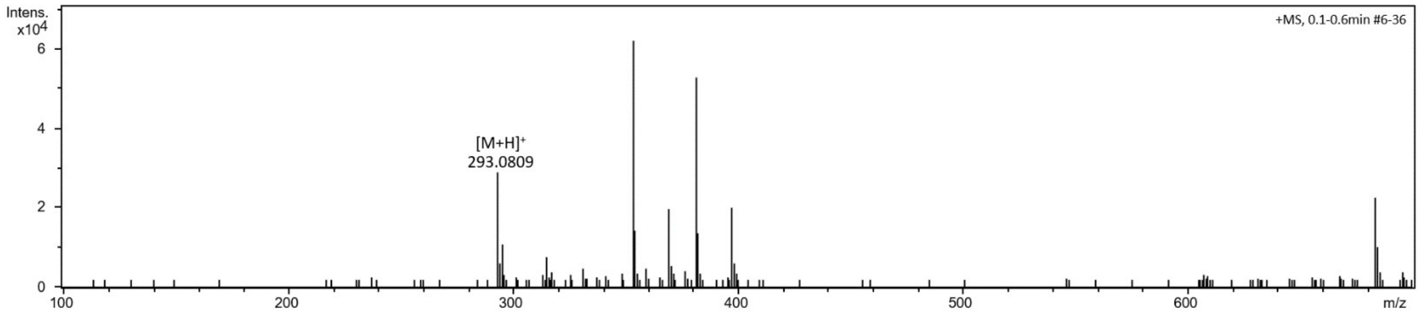

Figure S9. ESI-MS spectrum of compound MM7.

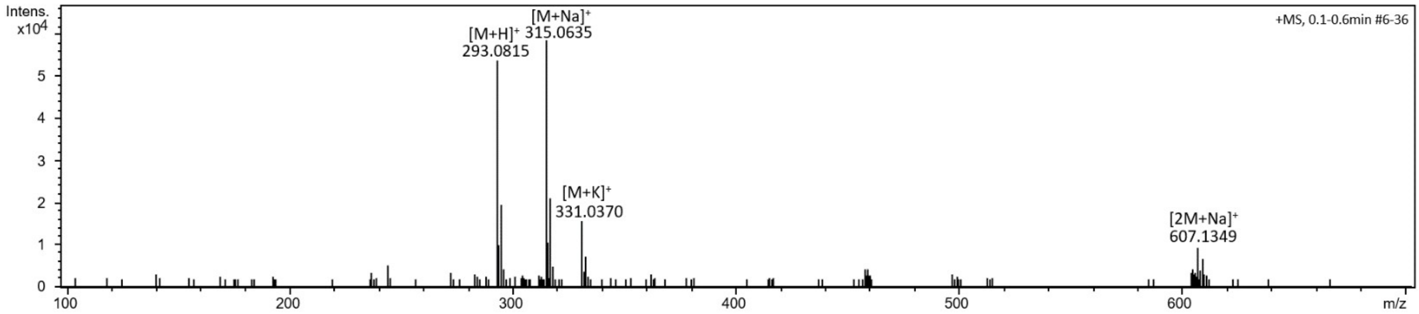

Figure S10. ESI-MS spectrum of compound MM8.

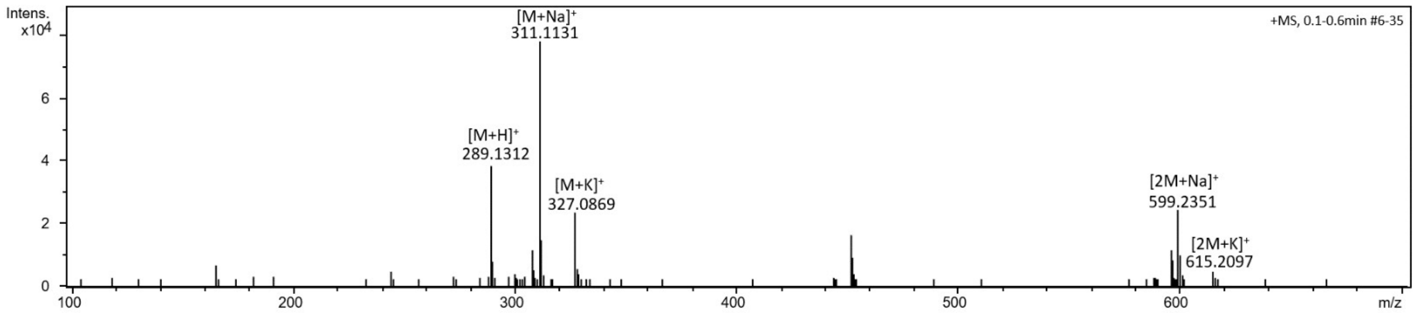

Figure S11. ESI-MS spectrum of compound MM9.

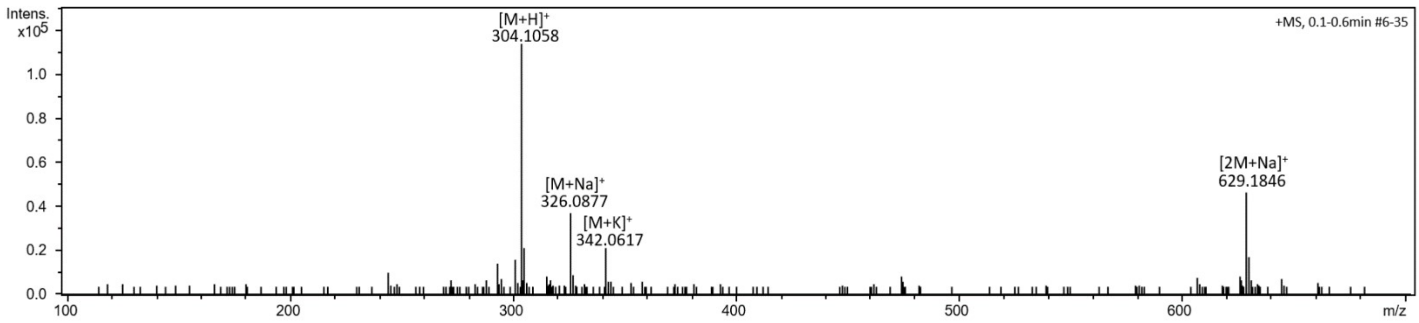

Figure S12. ESI-MS spectrum of compound MM10.

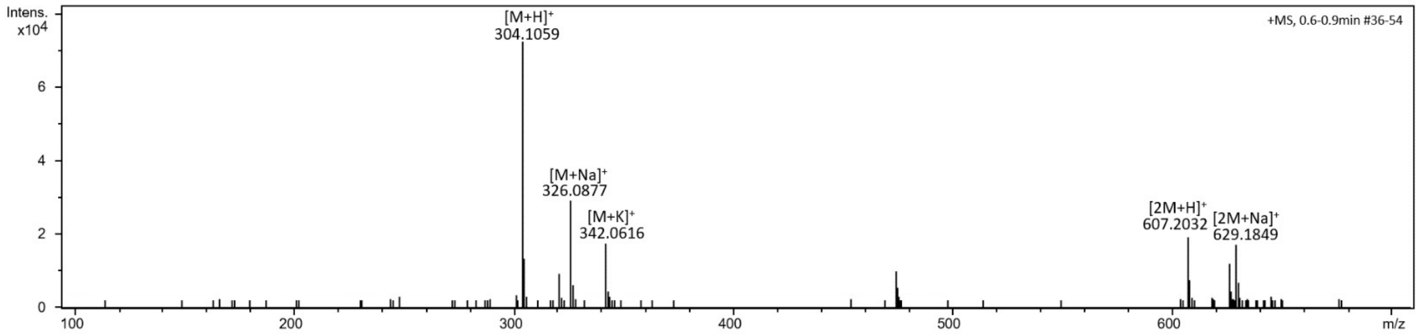

Supplement: Supplementary file 1 [file molecules-23-01545-s001.zip › molecules-321820-Sup-to publish/molecules-321820-Sup-to publish 1.pdf]
